# Real-time record of particulate matter along traffic roads and health risk assessment

**DOI:** 10.1007/s11356-026-38035-w

**Published:** 2026-07-08

**Authors:** Beata Górka-Kostrubiec, Tomasz Werner

**Affiliations:** Institute of Geophysics, Polish Academy of Sciences, Warsaw, Poland

**Keywords:** Traffic-pollution, Air pollution, Particulate matter grain-size fractions, Clean transport zone, Health effect

## Abstract

Air pollution from road traffic is a major environmental and public health concern in European cities. Many municipalities have introduced Clean Transport Zones (CTZs) to restrict high-emission vehicles and improve air quality. This study evaluated whether the planned CTZ in Warsaw, Poland, could reduce particulate matter (PM) concentrations and the associated respiratory exposure. A mobile monitoring system equipped with an optical particle analyzer measured real-time concentrations of four PM fractions along traffic routes, while the multiple-path particle deposition model was applied to assess age-dependent inhalation and deposition in the human respiratory tract. Average PM2.5 and PM10 concentrations did not differ significantly between the CTZ and the surrounding access routes. The highest concentrations occurred near major intersections, traffic lights, pedestrian crossings, and congestion zones, where PM2.5 and PM10 levels increased by up to an order of magnitude compared to uninterrupted traffic flow. Traffic disruptions, rather than CTZ location, are the primary drivers of short-term PM exposure. PM10–PM2.5 contributed approximately three times more to peak PM10 concentrations than under traffic-background conditions. Respiratory exposure analysis revealed significant age-related differences. Inhaled doses of PM1 decreased with age, deposition of PM10 in tracheobronchial and pulmonary regions was substantially higher in young children. PM10 mass deposition per area in 3-year-old children (0.78 μg m⁻^2^) was over 37 times higher than in adults, indicating a considerably greater respiratory surface burden. Improving air quality in Warsaw requires not only restricting older vehicles but also reducing traffic congestion and maintaining smooth vehicle flow to limit emissions of PM.

**Supplementary Information:**

The online version contains supplementary material available at 10.1007/s11356-026-38035-w.

## Introduction

In an effort to mitigate the adverse effects of air pollution from road traffic, many European city authorities have introduced Clean Transport Zones (CTZ) or Low Emission Zones (LEZ). These zones require vehicles entering them to meet specific exhaust emission standards, commonly referred to as the Euro standards, which are linked to the vehicle’s year of manufacture. There are currently more than 320 such zones in Europe (https://cleancitiescampaign.org/research-list/lez-essential-guide/?utm_source=chatgpt.com). The most extensive Low or Ultra Low Emission Zone system is located in London, the capital of the United Kingdom. The London-wide LEZ scheme has proven highly effective in reducing the number of older, more polluting vehicles. A few years after its implementation, measurements showed a reduction of about 44% in the average concentration of harmful NOₓ in the city area. Meanwhile, in the city center, the average concentration of PM2.5 (particulate matter with a grain size of less than 2.5 µm) decreased by approximately 9%, and in some locations by as much as 26% (AQMR 2021).

In Warsaw, CTZ zone regulations went into effect on July 1, 2024. The CTZ rollout comprises five stages, extending from 2024 to January 1, 2032, with progressively stricter limits on vehicle emissions within the zone. The zone covers approximately 7% (37 km^2^) of Warsaw’s total area, encompassing the main city center. At present, there is no comprehensive car tracking system, and enforcement by city authorities remains limited. Under the current regulations, cars owned by Warsaw taxpayers and purchased before 2024 are exempt from the CTZ requirements until 2027. The same exemption applies to vehicles owned by seniors born before 1956 and to antique cars. Moreover, the regulations for heavier vehicles (buses and trucks exceeding 3.5 tons) are less stringent than those for passenger cars. Occasional entry of older vehicles into the zone is also permitted, up to four times per year. In the first stages of the process – stages 1 and 2 from July 2024 until 31.12.2027, the majority of cars (around 97% as estimated by city officials) will be able to travel in CTZ without any limits. From January 2028, gasoline cars older than 23 years and diesel cars older than 14 years will be banned that may limit the number of older cars travelling in the CTZ. According to city projections, by 2032 when the zone is fully imposed, the CTZ will lead to an 80% reduction in NOₓ emissions and a 69% reduction in PM emissions.

The removal of older combustion vehicles from the most congested areas aims to reduce local air pollution, as gasoline and diesel vehicles are known to contribute significantly to pollution levels, particularly at intersections and along major roads (Kecorius et al. 2019). Diesel vehicles are a major source of PM, which consists of soot and a variety of toxic organic compounds (Mendoza-Villafuerte et al. 2017; Alves et al. 2023). Due to their chemical and physical properties, as well as their low mass, fine particles from motor vehicle emissions can penetrate the deeper regions of the lungs, where they may exert cytotoxic and even carcinogenic effects on humans (Park et al. 2018; Bendtsen et al. 2020; Giechaskiel et al. 2022).

There is substantial and growing evidence linking exposure to air pollution with a range of adverse health outcomes across all stages of life (Becker et al. 2003; Lippmann and Chen 2009; Campbell et al. 2014; Mannucci et al. 2015; Jørgensen et al. 2016; Kheirbek et al. 2016; Harrison et al. 2017; Bazyar et al. 2019; Fongsodsri et al. 2021). Numerous epidemiological studies have shown a correlation between PM exposure and increased morbidity, depending on the specific mass and size of PM fractions (Liu et al. 2013; Apte et al. 2015; Chen et al. 2015; Hassanvand et al. 2015; Kim et al. 2015; Samek et al. 2018; Caggiano et al. 2019). The wide range of health effects associated with PM exposure includes respiratory diseases (Luong et al. 2017; Yoshizaki et al. 2017), cardiovascular diseases (Nyhan et al. 2014; Wu et al. 2022), cerebrovascular effects (Alexeeff et al. 2021), low birth weight (Kim et al. 2016; Han et al. 2018; Chang et al. 2025), DNA mutations (Lei et al. 2018; Quezada-Maldonado et al. 2021), and kidney diseases (Bowe et al. 2018), among others.

Mobile monitoring has emerged as a critical and widely adopted approach for assessing small-scale spatial variations in traffic-related PM exposure in urban areas (Gozzi et al. 2016). Depending on the specific objectives of a given study, different sampling strategies are applied to evaluate traffic emissions and the associated health risks. Traditional approaches often integrate stationary air quality monitoring with numerical modeling to estimate PM concentrations over large urban areas, as demonstrated by Ginzburg et al. (2015). However, fixed-site monitoring networks have limited ability to capture fine-scale spatial gradients in pollutant concentrations, particularly in areas heavily influenced by local traffic emissions. Consequently, mobile monitoring systems have received significant attention because of their ability to provide high-resolution spatial data and identify localized pollution hotspots. Previous studies have highlighted the potential of mobile platforms to characterize intra-urban PM variability and the influence of traffic intensity and urban morphology on exposure patterns. For example, Merbitz et al. (2012) combined mobile measurements with regression modeling to study the distribution of PM in urban areas. Furthermore, Patton et al. (2014) demonstrated strong micro-scale variability in PM concentrations near major roadways by using mobile monitoring in communities adjacent to highways. Methodological advances proposed by Van den Bossche et al. (2015) further emphasized the importance of repeated measurements and temporal representativeness in producing reliable urban air pollution maps.

More recent studies have confirmed that using mobile monitoring systems—such as bicycles equipped with optical particle spectrometers—is an effective approach for measuring ambient PM concentrations near roadways (Cipoli et al. 2022). Simonen et al. (2019) characterized real-driving emissions from gasoline and diesel passenger cars in terms of fresh particle generation and secondary aerosol formation. In real-driving studies, particle number emissions during typical driving were found to be, on average, 4.8 times higher than those recorded in laboratory tests. This discrepancy may be attributed to more effective nucleation processes occurring when emissions are diluted in real, polluted, and humid ambient air (Feng et al. 2018). These findings highlight the importance of combining mobile monitoring techniques with real-driving emission analyses to improve the evaluation of urban PM exposure and to better understand the complex spatial and temporal dynamics of traffic-related air pollution.

Despite significant advances in mobile air pollution monitoring and traffic-related exposure assessment, a significant research gap remains in linking high-resolution exposure measurements with health risk assessment. Previous studies have focused on characterizing the spatial variability of PM concentrations using stationary or mobile monitoring systems or evaluating the health implications of air pollution across broader spatial scales. However, conventional stationary monitoring networks are inadequate for evaluating short-term, localized exposure peaks associated with traffic emissions, which can significantly impact public health. Consequently, comprehensive approaches that integrate continuous mobile monitoring, high-resolution exposure mapping, and health risk assessment frameworks are required. This strategy is critical when implementing Clean Air Zones and other urban emission-control programs because accurate identification of pollution hotspots and population exposure patterns is essential for evaluating the effectiveness of mitigation measures and supporting evidence-based urban air quality management policies.

The objective of this study was to determine the real-time concentration of PM directly at the source of emission along traffic routes, including the designated CTZ and selected major access roads to the zone. A mobile monitoring system equipped with an optical PM concentration analyzer was installed in an electric vehicle. The vehicle traveled along the bus lane, which is in proximity to moving vehicles such as cars, motorcycles, buses, and trucks, as well as cars equipped with modern exhaust emission reduction systems.

The present study provides a pre-implementation baseline of traffic-related PM concentrations and respiratory exposure in Warsaw. These results will serve as a critical reference dataset for future assessments of the effectiveness of the CTZ after its implementation.

The objective of this study was also to quantify the deposition of PM fractions to elucidate the impact of pollution across different age groups (children and two categories of young people) and within specific regions of the human respiratory system—namely, the extrathoracic, tracheobronchial, and pulmonary regions. This approach is consistent with previous research by Kecorius et al. (2019) and Lv et al. (2021). The widely recognized Multiple Path Particle Dosimetry (MPPD) model was employed to estimate total deposition, regional deposition, and lobar deposition fractions per airway generation. Based on our understanding of the PM fractions emitted by vehicles, we were able to estimate the inhaled dose of particles for pedestrians moving through the study area, particularly near major roads. We also identified the regions of the human respiratory system where specific PM fractions tend to deposit and linked these findings to potential adverse health effects in humans.

## Materials and methods

### Description of sampling location

Warsaw, the capital of Poland and the most populous Polish agglomeration, is located in the center of the country on the Masovian Plain in the Central European region. The city lies along the Vistula River, which divides it into two distinct parts. Administratively, Warsaw comprises 11 districts on the right bank of the Vistula River and 7 districts on the left bank. The city’s central business district, known for its modern skyline dominated by glass skyscrapers, adjoins several historic quarters, creating a distinctive blend of contemporary and traditional architecture. Warsaw serves as the seat of numerous state institutions, including the parliament and the president’s office, as well as the headquarters of many prominent Polish companies. The city has a population exceeding 1.7 million, with a substantial number of people from surrounding areas commuting to Warsaw for work, shopping, or academic purposes. The Warsaw agglomeration has a well-developed public transportation system, consisting of buses, trams, an underground metro, and an Urban Rapid Railway network.

According to data from the European Automobile Manufacturers Association (ACEA), Poland has 747 vehicles per 1000 inhabitants—placing it second among European Union (EU) member states in terms of vehicle density. The total number of registered vehicles in Poland exceeds 24.3 million, ranking the country fifth among EU members in this regard. However, despite the high number of vehicles in use, Poland ranks lowest in the EU concerning the average age of vehicles. ACEA data indicate that the average age of vehicles on Polish roads exceeds 14 years, compared to the EU average of 11.5 years. In terms of fuel type, 53.2% of vehicles in Poland are powered by petrol engines, while 31.6% use diesel engines.

**Fig. 1:**
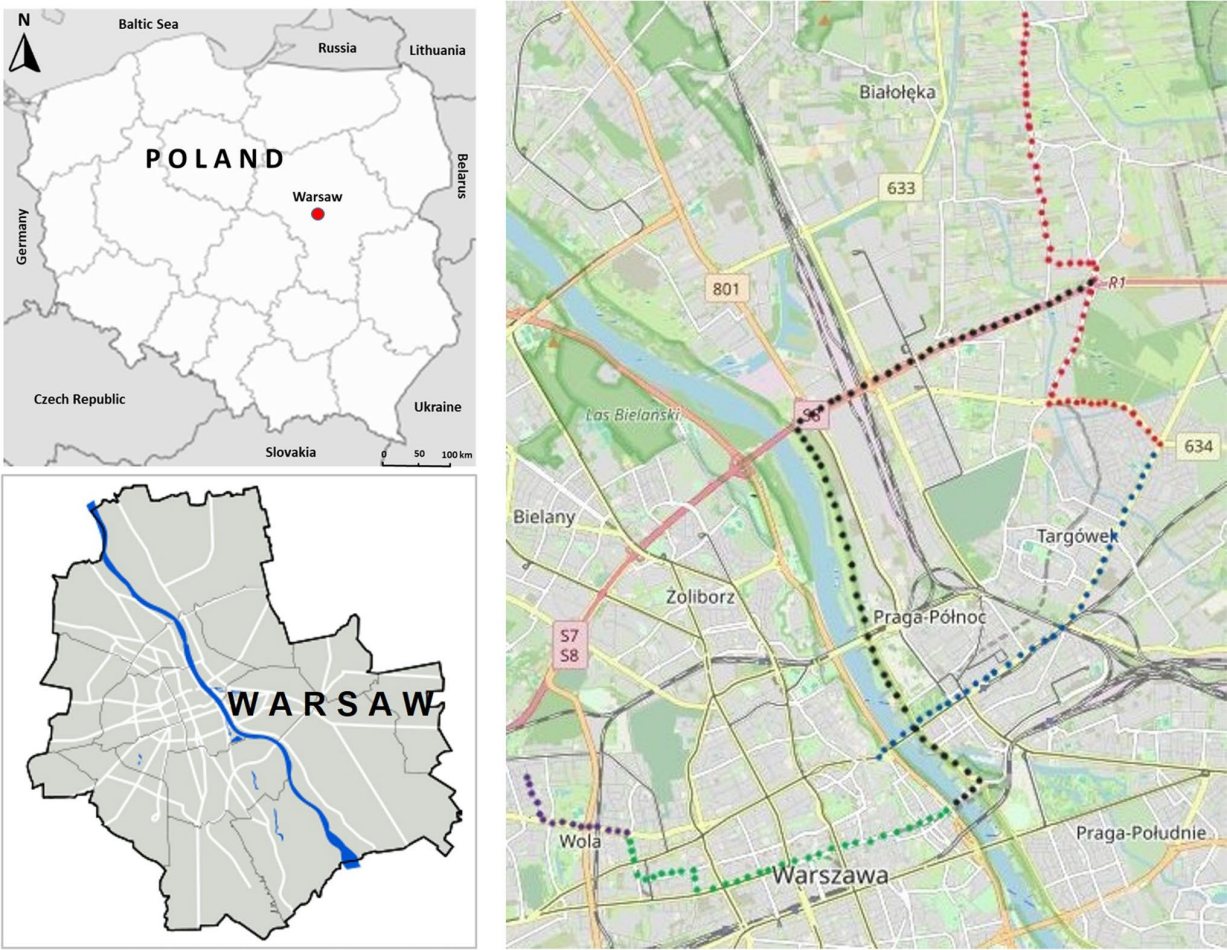
Sampling sites and locations of the studied areas. Green dots indicate roads in Area 1 (Jerozolimskie Av., Prosta-Żelazna St., Chmielna-Towarowa St.) located within the Clean Transport Zone (CTZ), blue and black dots indicate roads in Area 2 (Jagiellońska St., Wyb. Szczecińskie St., Radzymińska St.), violet dots indicate road in Area 3 (Górczewska St.) and red dots indicate roads in Area 4 (Ostródzka-Głębocka St.) Source: Base map © OpenStreetMap contributors (www.openstreetmap.org)

The mobile monitoring campaigns were conducted over 3 months, from May 23 to July 25, 2023. This period corresponds to the summer season, when road traffic constitutes the predominant source of pollution. During this season, the influence of low emissions and those originating from municipal heating plants is relatively insignificant. The lack of major industrial activity in Warsaw and its surrounding areas further supports this observation. Consequently, during the study period, in addition to traffic-related sources, long-range sources were also considered potential contributors to air pollution levels in the city. To account for temporal variations in emissions, measurement campaigns were carried out during the morning and evening rush hours on five weekdays, when traffic intensity is higher than at other times of the day. This pattern is associated with the morning commute of employees to business and administrative centers—primarily located in the city center—and their return to residential areas in the afternoon. The research route included roads connecting the CTZ with a low-rise residential district (Wschodnia Białołęka) located 16 km to the north, as well as an access road leading to the CTZ from the west. The total length of the monitoring route was 28 km. Figure 1 presents the route with corresponding street names and the investigated areas.

The route was divided into four areas, with individual roads assigned to each.

The first area (Area 1) is located within the CTZ and includes Jerozolimskie Avenue, Prosta Street, Chmielna Street, Żelazna Street, and Towarowa Street. These streets traverse the city’s central business district, characterized by a dense concentration of high-rise buildings, primarily skyscrapers. Jerozolimskie Avenue and Towarowa Street are wide thoroughfares, each consisting of three lanes in both directions, with a tram line running along the median that separates opposing traffic flows. These major arteries intersect with numerous cross streets, forming some of the city’s largest intersections. Many of these intersections include roundabouts and multiple pedestrian crossings regulated by traffic lights. In contrast, Prosta, Chmielna, and Żelazna Streets are relatively narrow, with one or two lanes in each direction. These streets are located in an area with a high density of buildings, producing a distinct urban canyon effect. They also experience relatively high traffic volumes (Table S1). The tables and figures referenced as S1, S2, and so on are provided in the supplementary material.

The second area (Area 2) includes Radzymińska Street, Wybrzeże Szczecińskie Street, and Jagiellońska Street, located on the right bank of the Vistula River. These major thoroughfares serve as key connectors linking the northern districts of Warsaw and neighboring towns outside the city limits with the city center. They are characterized by heavy traffic, particularly during the morning and evening rush hours (Table S1).

The third area (Area 3) encompasses Górczewska Street, a major thoroughfare characterized by its wide layout and two lanes in each direction. This route connects the southern districts of Warsaw with the communities located on the southern outskirts of the city center. Area 3 exhibits traffic conditions comparable to those observed in Area 1.

The fourth area (Area 4) includes Ostródzka Street and Głębocka Street, located in the Wschodnia Białołęka district, north of the city center. These thoroughfares serve as the primary routes for district residents commuting to workplaces, educational institutions, and childcare facilities in the city center. Both roads are relatively narrow, with a single lane for vehicular traffic in each direction. This limitation, combined with the presence of numerous intersections and roundabouts, contributes to the complexity and congestion of traffic patterns in the area.

### Measurement protocol and instruments

The dataset comprised seven sampling sessions conducted on May 23, July 3–4, July 18, and July 25, 2023. Three morning sessions were carried out between 7:00 and 8:30 a.m., corresponding to the morning rush hour, while four afternoon sessions were conducted between 4:00 and 6:00 p.m., corresponding to the afternoon rush hour. To minimize potential variability in vehicular traffic, all sessions began at the same sampling point and approximately the same time of day. Each session lasted about 1.5 h. All measurement days were characterized by clear skies and the absence of precipitation. Meteorological parameters—including average temperature, pressure, humidity, and precipitation amounts for each day and hour—are presented in Table S2.

Measurements were performed using an optical DustTrak DRX 8533 aerosol monitor (TSI, USA), equipped with a class 1 laser and a 90° light-scattering sensor. The DustTrak was placed on the front passenger seat of an electric vehicle. A rubber tube was used to extend the air intake to the aspirator; the tube exited through an open window and was secured to the vehicle body with tape in a parallel orientation to the direction of travel, ensuring unobstructed airflow into the aspirator. The air inlet of the rubber tube was positioned 1.2 m above the road surface. The DustTrak is capable of measuring mass concentrations of PM with aerodynamic diameters ranging from 0.1 to 15 μm, with a measurement range of 0.001 to 150 mg/m^3^. The instrument’s precision, defined as measurement accuracy, is 0.1% of the reading. The mass concentrations of four distinct PM fractions—PM1 (0.1–1 μm), PM2.5 (0.1–2.5 μm), PM4 (respirable fraction, 0.1–4 μm), and PM10 (0.1–10 μm)—were recorded simultaneously with a time resolution of one second. PM-laden air was continuously drawn into the optical chamber at a total flow rate of 3 L/min using an internal pump. Before use, the PM monitor was calibrated in accordance with ISO 12103-1 standards, employing a standardized A1 dust sample (Arizona Dust; TSI) provided by the service company. A zero calibration was performed prior to each measurement using a zero filter attached to the instrument.

Each measurement interval lasted 5 s and was continuously repeated throughout the vehicle’s movement along the selected route. The selected route included streets with designated bus lanes that allowed the operation of electric vehicles. This enabled the electric vehicle to travel freely and maintain a relatively constant speed along most sections, even when vehicles in adjacent lanes were moving slowly or were subject to congestion. The geographic position and speed of the vehicle were recorded using the GPS Logger app (https://gpslogger.app/) installed on a smartphone. Measurement of PM concentration and position began and ended simultaneously. After each monitoring session, the data obtained from the application—comprising position (latitude and longitude), time, and speed—were correlated with the recorded PM fraction concentrations using the time variable as a reference.

To determine the real-time distribution of PM along roads, the analysis focused on PM10 and PM2.5 fractions. These fractions were selected because they are routinely measured in mobile and stationary monitoring networks, enabling direct comparison of datasets. PM10 and PM2.5 concentration data from five stations of the Warsaw Air Quality Monitoring Network (Table S3), operated by the Inspectorate for Environmental Protection, were used as a reference dataset for this purpose. This approach enabled us to assess the consistency and representativeness of mobile measurements in relation to established stationary monitoring observations.

### Multiple-path particle dosimetry model and inhaled dose

The deposition of PM fractions in the human respiratory tract (HRT) was calculated using the Multiple-Path Particle Dosimetry (MPPD) model. The MPPD model software is publicly available for free download (http://www.ara.com/mppd/). Four primary deposition parameters—namely, deposition fraction (DF), deposition mass (DM), deposition mass per area (DMA), and deposition rate (DR)—were calculated for the inhaled PM1, PM2.5, PM4, and PM10 fractions within the HRT. The deposition fraction is defined as the ratio of the number of particles of a specific size deposited in a given region of the HRT to the number of particles of the same size entering the airways (Manojkumar et al. 2019). The deposition mass represents the mass of PM deposited in a specific region of the HRT. The deposition density (DDP) is the DM normalized by the surface area of the lungs, while the deposition rate indicates the rate at which PM mass accumulates in the respiratory tract.

Lung particle deposition was simulated using the Age-Specific 5-Lobe model, applying two geometric configurations of lung structure representative of those in three-year-old children and 21-year-old young adults. The Yeh–Shum 5-Lobe model was used to simulate lung deposition in adults aged 25–30 years. The physiological parameters used for each age group—such as functional residual capacity, upper respiratory tract volume, breathing frequency, and tidal volume—are presented in Table S4. These parameters conform to the recommendations of the International Commission on Radiological Protection (ICRP 1994) and the MPPD model.

In the MPPD model, the PM deposition fraction is calculated using the theoretically derived deposition efficiencies of various mechanisms such as diffusion, impaction, and sedimentation (Anjilvel and Asgharian 1995). This model provides flexibility to use density, aspect ratio, and PM diameter for calculation. The diameter can be specified as the count median diameter, mass median diameter, or mass median aerodynamic diameter. Instead of specifying a particular particle size, the multiple particle sizes, i.e., size-segregated PM concentrations can be provided. Deposition values for each particle size (PM10, PM2.5, PM4, and PM1) are then calculated using the count median diameter 10, 2.5, 4, and 1, respectively (Manojkumar et al. 2019). In reality, because ambient aerosols exhibit complex and variable size distributions, the estimated deposition fractions could be interpreted as approximations based on the assumed particle size characteristics.

In this study, the deposition parameters were estimated and visualized for the four size-segregated PM concentrations. While PM2.5 and PM10 are the most commonly used metrics in air quality and health assessments, the inclusion of PM1 and PM4 provides a more detailed characterization of particle size-associated health risks. PM1 represents the finest particle fraction capable of penetrating the deepest regions in the respiratory system and is often associated with combustion-related emissions. PM4 provides additional insights on particles depositing within the thoracic region of the respiratory tract, bridging the gap between PM2.5 and PM10. Therefore, assessing health risks across PM1, PM2.5, PM4, and PM10, instead of relying solely on PM2.5 and PM10, allows us to more comprehensively evaluate exposure and potential adverse health effects.

The short-term inhalation dose was calculated for each age category using the equation proposed by Yeh and Schum (1980):

Dose (μg) = $\sum_{i}{\mathrm{P}\mathrm{M}}_{\mathrm{i}}$  × DF × VE × t,(1)

where PM_i_ is the mean concentration of PM for the *i*-th size fraction (in μg/m^3^); DF is the deposition fraction of particles in different parts of the respiratory system (dimensionless), calculated using the MPPD model; VE is the ventilation rate per minute (in m^3^/min); and t is the exposure time. A mean VE value of 12.5 × 10⁻^3^ m^3^/min, as recommended by the US Environmental Protection Agency (U.S. EPA 2011), was applied to represent short-term exposure for children and adults aged 3–35 years during light-intensity walking. It was assumed that pedestrians walking at a light intensity breathe spontaneously through the nose, with an average walking duration of 30 min.

This study used version 3.1 of the MPPD software, which functions primarily as a deterministic model for assessing particle deposition within the respiratory tract. Most calculations are performed without an explicitly displayed iterative loop. In practice, MPPD 3.1 users typically do not manually specify the number of iterations; rather, they configure only the physical parameters and the breathing scenario. The model terminates its calculations upon reaching the convergence criterion.

## Results

### Concentration of PM fractions in potential clean transport zone areas and along access roads to the city center

**Fig. 2:**
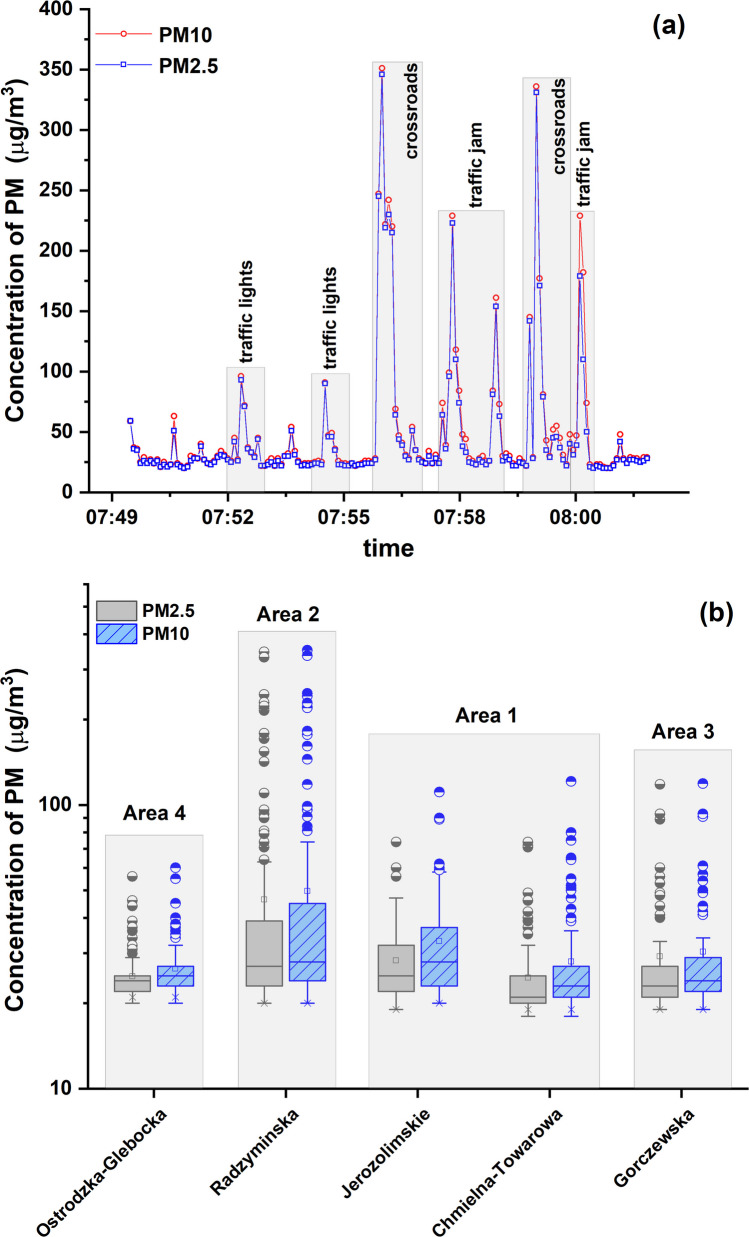
**a** Concentrations of PM10 (red line) and PM2.5 (blue line) along Radzymińska Street in Area 2. Data were collected on July 18, 2023, during the morning rush hour (7:50–8:02 a.m.). The maximum values of PM 10 and PM2.5 were around 350 μg/m^3^ (10-fold above the background level). The electric vehicle traveled 6 km in the bus lane. **b** Box-and-whisker plot showing PM2.5 and PM10 concentrations on roads in all study areas on the morning of July 18, 2023. Outliers for each traveled road segment are marked as half-filled circles

An illustrative example of in situ measurements of PM2.5 and PM10 concentrations is presented in Fig. 2a. The data were collected during the session conducted on July 18, 2023, in the morning rush hours along Radzymińska Street, located within Area 2; this area experiences heavy traffic, particularly during morning rush hours. For comparison, data collected along Jerozolimskie Avenue in Area 1 the city’s central business district with a dense concentration of high-rise buildings) on the same day during the afternoon rush hours are presented in Fig. S1a. The dataset reveals numerous peaks, with PM10 and PM2.5 concentration values reaching up to 350 and 345 μg/m^3^, respectively. An analysis of PM concentration in relation to geographic location showed that gradual increases and decreases (peaks) occurred primarily at major intersections with heavily trafficked access roads (see Fig. 2a). Some peaks, however, were attributed to the proximity of noncompliant vehicles near the electric car performing the measurements, as noted by the operator.

Peaks in the PM record were defined as values that exceeded the upper quartile of the PM2.5 and PM10 records for each road segment traveled during the morning or evening measurement session (see Table S5). For instance, on July 18, 2023, on Radzymińska Street (Fig. 2a), 37 of the 155 recorded values were peak values for PM2.5 and PM10. The mean value calculated for the entire recorded data set for PM2.5 was 46.34 µg/m^3^, and the Q3 quartile was 39 µg/m^3^. After eliminating the 37 peaks from the PM2.5 record, the mean value for the remaining data set decreased to 26.06 µg/m^3^. Furthermore, the mean values after removing the PM values that exceeded the Q3 threshold from the original record were considered traffic background values (e.g., 26.06 µg/m^3^ for the aforementioned PM2.5 record). This procedure was applied to each segment of the record, and the background was calculated independently for each measurement session. These values are listed in Table S5.

Figure 2b presents the box-and-whisker plot for the entire route traveled on July 18th, a.m. Mean values and outliers were calculated for each street segment across the four areas. In each dataset, several outliers were observed exceeding the upper quartile (0.75). Although the mean PM10 and PM2.5 concentrations were around 30 μg/m^3^, both the number and magnitude of outliers varied substantially between areas. A detailed analysis revealed that these outliers were primarily associated with peaks (Fig. 2a and Fig. S1a) in the PM concentration record. For the route traveled on July 18th a.m., a higher frequency of peaks related to local traffic congestion was observed along major roads in the city center (e.g., Jerozolimskie Avenue, Area 1) and in parts of Area 2 with heavier traffic. After removing peaks with values exceeding the upper quartile for individual roads within each area, the mean concentrations of PM10 and PM2.5 were 25.2 and 23.6 μg/m^3^, respectively. These values can be considered representative of the traffic background for that sampling day. For comparison, the average PM fraction values recorded by stationary monitoring stations in Warsaw on July 18, 2023, between 7:00 and 8:00 a.m. are provided in Table S3. The urban-traffic station reported PM10 and PM2.5 concentrations of 22.3 and 8.8 μg/m^3^, respectively, while the suburban-traffic station recorded values of 7.8 and 5.9 μg/m^3^ for PM10 and PM2.5, respectively.

**Fig. 3:**
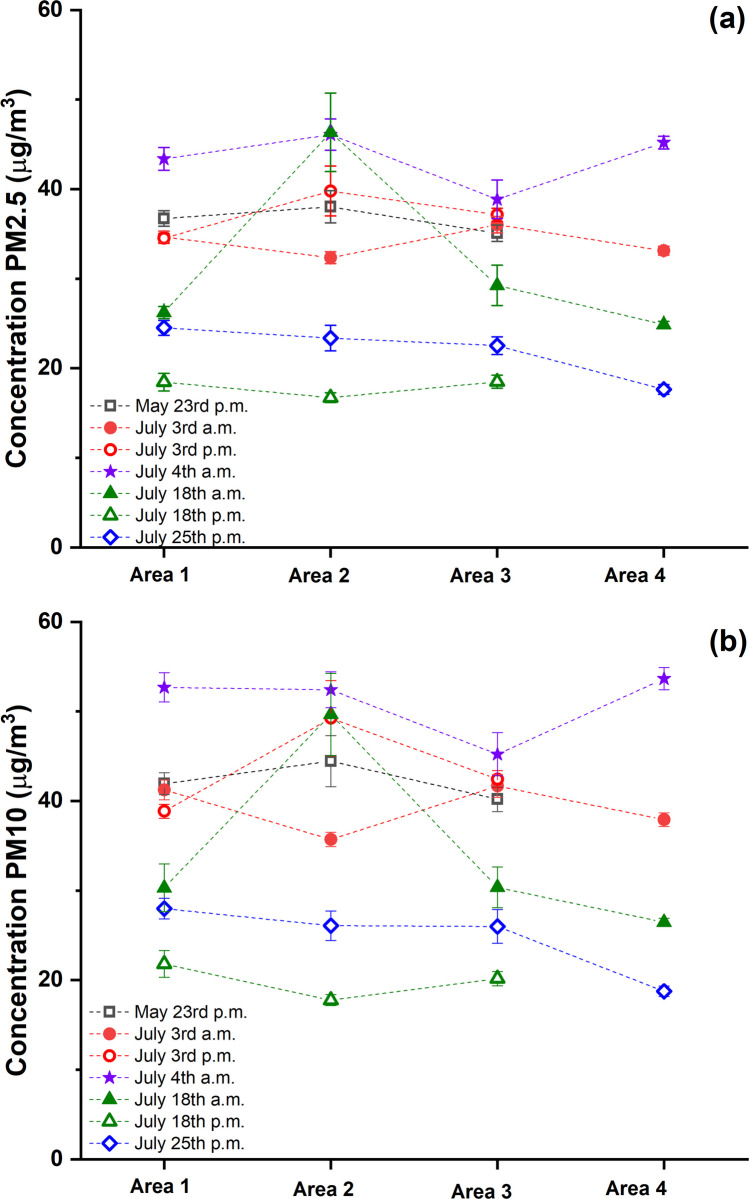
Average concentrations of **a** PM2.5 and **b** PM10 in individual areas: Area 1 within the current CTZ (Jerozolimskie Av., Prosta-Żelazna St., Chmielna-Towarowa St.), and Areas 2 (Jagiellońska St., Wyb. Szczecińskie St., Radzymińska St.), Area 3 (Górczewska St.), and Area 4 (Ostródzka-Głębocka St.), including access streets to the city center

Figure 3 presents the mean concentrations of PM10 and PM2.5, calculated from all data—including peak values—for individual areas located within the CTZ; Area 1) as well as those outside the zone (Areas 2–4). The dataset covers the period from May 23 to July 25 and is divided into morning and afternoon traffic peaks. Statistical parameters, including mean PM10 and PM2.5 concentrations, standard deviations (SD), and the number of peaks obtained from all seven measurement sessions, are provided in Table S5. In addition, the standard error of the mean (SE) was calculated for the mean PM10 and PM2.5 concentration values (see Table S5 for details) to determine whether the differences between the means values were meaningful. Interestingly, the concentrations of both PM fractions were more dependent on the day of measurement than on the specific area. No significant differences in PM concentrations were observed between roads in the city center with high traffic intensity and dense development, roads with moderate and high traffic intensity, and residential district roads. On average, the differences in concentration between areas did not exceed 20% for PM10 and 16% for PM2.5. However, during the sessions on July 18th a.m. and July 25th p.m., the differences between areas exceeded 60% and 30%, respectively, for both PM fractions. This variation resulted from the relatively high PM10 (50 μg/m^3^) and PM2.5 (46 μg/m^3^) concentrations recorded on the heavy traffic road in Area 2 on July 18th a.m., and the comparatively low PM10 (19 μg/m^3^) and PM2.5 (18 μg/m^3^) values recorded in Area 4 in the residential district on July 25th p.m.

The exceptionally high PM10 and PM2.5 concentrations in Area 2 on the morning of July 18 were likely due to numerous high-intensity peaks observed that day along Radzymińska Street (Fig. 2a). These atypical values were probably caused by major traffic congestion and the specific traffic conditions of the road. During the morning rush hour, all three lanes leading toward the city center are typically filled with vehicles, causing significant delays at intersections and traffic lights. In contrast, the relatively low PM concentrations recorded on July 25 can be attributed to the time of day. Vehicle intensity is generally lower in the afternoon than in the morning rush hour, as commuters returning home tend to depart over an extended period, whereas morning departures for work typically occur within a narrow time window between 7:00 and 8:30 a.m. On the other hand, mean PM2.5 concentrations varied considerably, ranging from 17 to 46 μg/m^3^ depending on the day of measurement (Fig. 3a). PM10 concentrations followed a similar pattern, ranging from 18 to 53 μg/m^3^ (Fig. 3b). In each study area, the highest mean concentrations of both PM fractions were recorded on July 4 during the morning peak traffic period. This finding corresponds with the highest PM2.5 and PM10 concentrations measured at the stationary urban-traffic station on July 4—21.4 μg/m^3^ (Fig. S2a) and 92.6 μg/m^3^ (Fig. S2b), respectively.

A significant part of the study involved determining the proportion of PM attributable to emissions from moving vehicles. The particle size distribution of airborne PM is broad, encompassing diameters from a few nanometers (UFP) to tens of micrometers (e.g., crustal particles). The size of primary particles emitted by modern combustion engines typically ranges from approximately 10 to 100 nm (Giechaskiel 2020). According to recent data from the European Environment Agency (EEA 2020), a substantial fraction of road transport–related PM is attributed to nonexhaust sources such as brake, tire, and road surface wear—accounting for 33.9% of PM10 and 26.7% of PM2.5 emissions. For each measurement point, the percentage contribution of PM with an aerodynamic diameter between 2.5 and 10 μm (PM10–PM2.5) to total PM10 was calculated. It was observed that data points with relatively high proportions of the PM10–PM2.5 fraction also corresponded to exceptionally high PM10 values (peaks). Consequently, the dataset was divided into two categories: (i) data with a high contribution of the PM10–PM2.5 fraction, corresponding to PM peaks, and (ii) the remaining data, described as traffic background. This approach aligns with previous observations for Radzymińska Street (Fig. 2a) and Jerozolimskie Avenue (Fig. S1), both streets with heavy traffic. The peaks were primarily attributed to PM emissions occurring during local traffic jams, where statistically more noncompliant vehicles are present. Within these peaks, the average percentage contribution of the PM fraction between 2.5 and 10 μm to PM10 was 24.4%, whereas for the traffic-background data, this contribution was 8.4% (Table S6). The percentage contributions of the PM2.5 fraction to PM10 for peak and traffic-background data were 75.6% and 91.6%, respectively (Table S6).

**Fig. 4:**
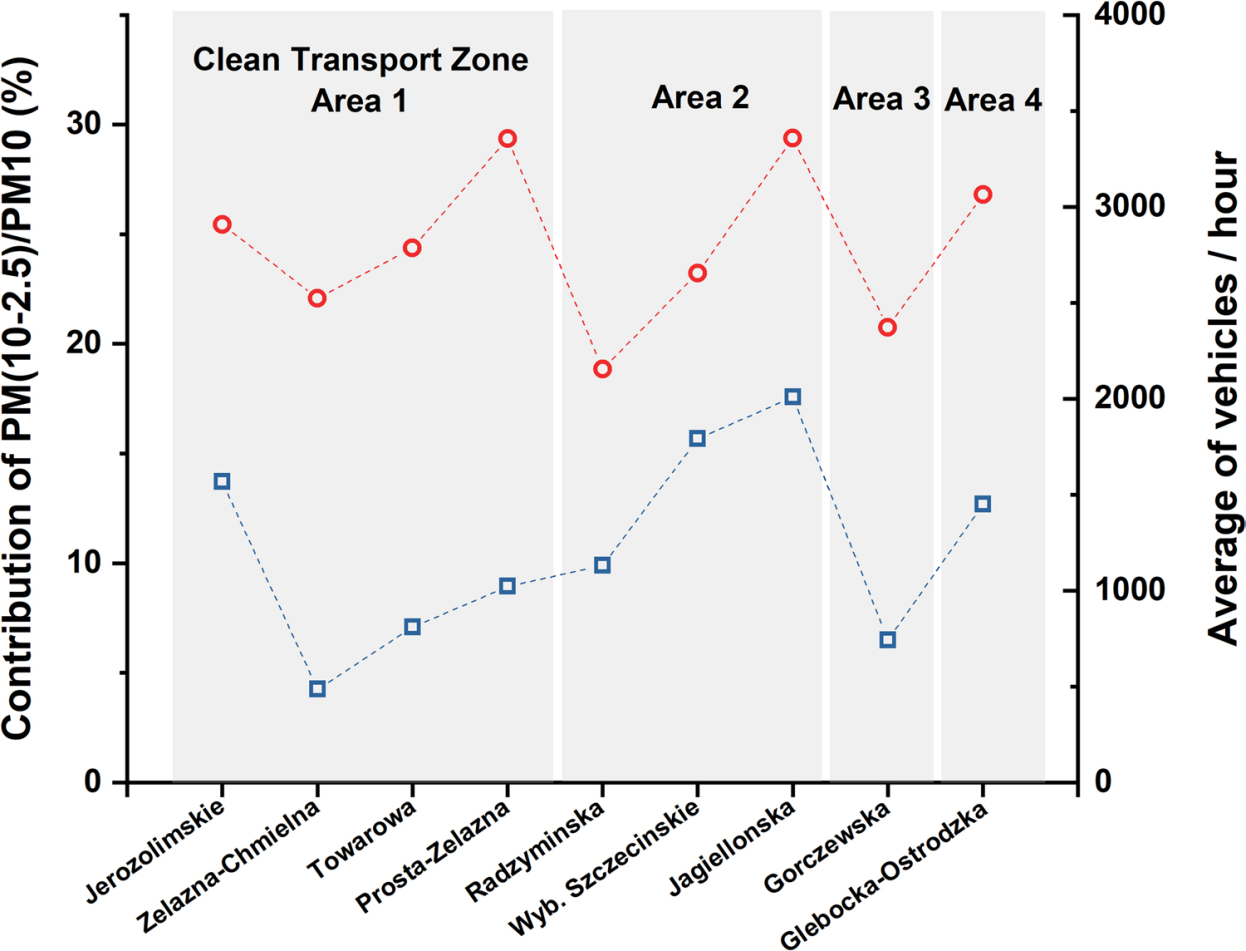
Average percentage contribution of the PM fraction with particle sizes between 2.5 and 10 μm (PM10–PM2.5) to PM10, determined for peak data (red open circles), and mean vehicle traffic intensity measured during morning and afternoon rush hours (blue open squares)

The percentage contributions of (PM10–PM2.5)/PM10 determined for individual streets across all areas are illustrated in Fig. 4. Figure 4 also shows mean vehicle traffic intensity during morning (7:00–8:30 a.m.) and afternoon (4:00–6:30 p.m.) rush hours, measured at automatic monitoring points between May and October 2022. These data were obtained from the Warsaw Municipal Roads Authority portal (https://zdm-warszawa.maps.arcgis.com/apps/dashboards/fb6da3d215334a489709f7dc02726311). A positive correlation was found between the contribution of (PM10–PM2.5) to PM10 and vehicle traffic intensity, with a Pearson correlation coefficient of 0.48. The average percentage contributions of (PM10–PM2.5) to PM10 calculated for streets within the current CTZ did not differ significantly from those determined for suburban roads (Głębocka and Ostródzka Streets) or the access routes to the city center.

### Accumulation of PM in the respiratory tract of selected age groups of humans

**Fig. 5:**
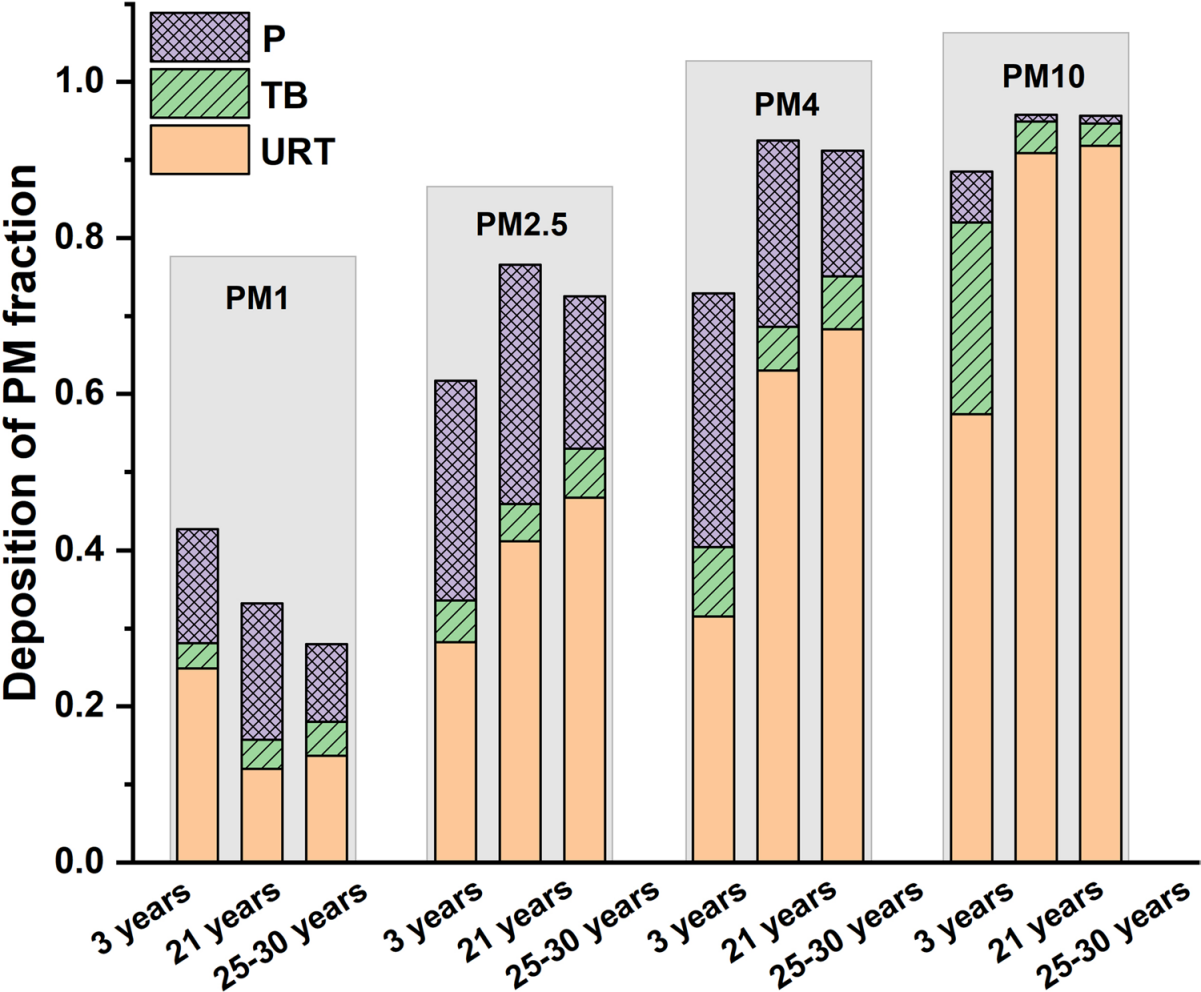
Deposition of PM1, PM2.5, PM4, and PM10 in specific regions of the human respiratory tract (upper respiratory tract, URT; tracheobronchial, TB; and pulmonary, P) for three age groups (3-year-old children, 21-year-old young adults, and 25–30-year-old adults)

The total and region-specific deposition parameters in the HRT for each age category are presented in Fig. 5 and listed in Table S7, while the deposition mass, deposition mass per area, and deposition rate are shown in Table S8. The deposition parameters were calculated using the average concentrations of PM fraction obtained for Area 1, which is located within the planned CTZ. To simulate the maximum exposure to PM, the deposition was determined for the PM concentration obtained on July 4th a.m. Simulations conduced for values deviating from the average concentrations PM did not reveal significant differences in deposition across HRT regions and age categories.

A substantial proportion of inhaled PM10 is deposited in the human airways, with a range of 89–96%. This is followed by PM4 (73–93%), PM2.5 (62–77%), and PM1 (28–43%). In consideration of the age-specific category, the total depositions of PM10, PM4, and PM2.5 were found to be higher for young people (21 years of age) and adults (25–30 years of age) than for children aged three. With regard to the smallest fraction (PM1), the highest deposition was observed in the HRT of 3-year-old children. This phenomenon may be attributed to differences in lung morphology between children and adults (Nag et al. 2005). The regional-specific deposition of PM1, PM2.5, and PM4 displayed a consistent pattern, with the highest deposition occurring in the URT, followed by the P and TB regions (URT > P > TB). For the PM10 fraction, however, deposition values were higher in the TB region than in the P region, following the pattern URT > TB > P. The deposition of PM10 in the TB (25%) and P (7%) regions of the HRT was markedly higher in 3-year-old children than in 21–30-year-old adults (TB—4%, P—1%).

According to the MPPD model, the respiratory tract consists of 28 generations of branching airways. This branching system begins at the trachea, continues through the bronchi and bronchioles, and terminates in the alveoli, where gas exchange occurs. The mean concentrations of PM10, PM4, PM2.5 and PM1 (see Tab. S7) were used to calculate the deposition parameters of each PM fraction across age groups, specifically from the 1 st to 16th generations of the respiratory tract—that is, within the tuberculosis region. The maximum mass deposition of PM10 in the TB region for children aged 3 years (9.24 × 10⁻^5^ µg) was nearly five times higher than that for young adults aged 21–30 years (1.38–1.88 × 10⁻^5^ µg) (Table S8). The maximum deposition rate values for PM1 and PM2.5 were more than twice as high in the 21–30-year age group (5.42–5.71 × 10⁻^4^ µg/min for PM1 and 4.56–5.32 × 10⁻^4^ μg/min for PM2.5) compared with the 3-year-old group (1.61 × 10⁻^4^ μg/min and 2.10 × 10⁻^4^ μg/min, respectively). In contrast, the maximum deposition rate values for PM4 and PM10 were similar across all age groups, ranging from 3.04–4.66 × 10⁻^4^ μg/min for PM4 and 1.65–2.63 × 10⁻^4^ μg/min for PM10. The deposition mass per unit area exhibited substantial variability, likely due to differences in airway geometry between children and adults. The lowest DMA values were recorded for the 21-year-old group, ranging from 0.021 to 0.033 µg/m^2^. In this age group, DMA values increased with decreasing PM particle size. A similar trend was observed for the 25–30-year-old group; however, in this case, DMA values ranged from 0.019 µg/m^2^ for PM10 to 0.058 µg/m^2^ for PM1. Conversely, in the 3-year-old group, the trend was reversed—DMA values increased with increasing PM particle size, reaching a maximum of 0.781 μg/m^2^ for PM10. This value was an order of magnitude higher than that observed for PM4 in the same age group.

**Fig. 6:**
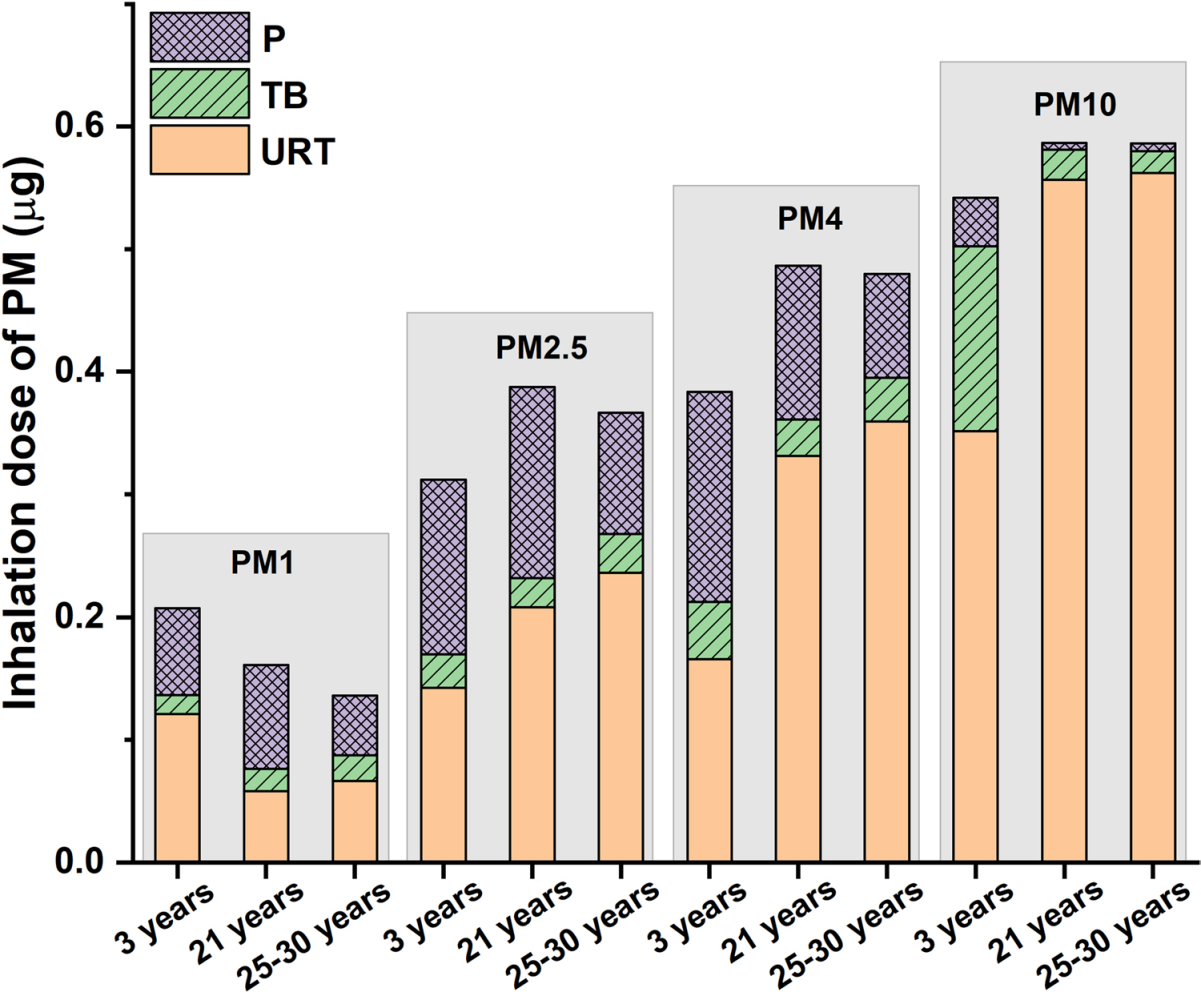
Inhalation doses of PM1, PM2.5, PM4, and PM10 in specific regions of the human respiratory tract (URT, TB, and P) for three age groups (3-year-old children, 21-year-old young adults, and 25–30-year-old adults). Inhalation dose was calculated for a 30-min exposure period during light-intensity activity (walking)

For each age group, the deposition of inhaled doses of all studied PM fractions in specific regions of the HRT (URT, TB, and P) was calculated for a 30-min exposure during a light-intensity walk (Fig. 6). Regarding the deposition of inhaled doses in specific regions, it was observed that the respirable fractions (PM1, PM2.5 and PM4) contributed the least to deposition in the TB region. A distinct difference in PM₁ deposition patterns was noted between age groups: the 21-year-old cohort exhibited a distribution pattern of P > URT > TB, whereas the 25–30-year-old adults showed URT > P > TB. For 3-year-old children, a slightly smaller proportion of the total mass of PM1, PM2.5, and PM4 was deposited in the P region (34–46%) than in the URT region (43–58%) during a 30-min walk. The coarse PM10 fraction exhibited a distinct deposition pattern, which remained consistent across all age groups, with deposition following the order URT > TB > P. In the 21–30-year-old group, nearly 95% of the total PM10 dose was deposited in the URT, whereas in the 3-year-old group, 65% was deposited in the URT, 28% in the TB region, and 7% in the P region.

## Discussion

### Characteristics of the PM2.5 and PM10 for the studied areas

The PM10 and PM2.5 records along the traveled routes exhibited substantial variation in the number of peaks, which were identified at major crossroads and were influenced by traffic intensity and the presence of older vehicles (Fig. 2a and Fig. S1). More than three consecutive peaks were generally associated with slow-moving traffic approaching major intersections, particularly in Areas 1 and 2 along the heavy traffic roads. Some peaks may also have resulted from the passage of individual older cars that did not meet emission standards. Analysis of the mean PM2.5 and PM10 concentrations for each session day (Fig. 3 and Table S5) showed minor differences between areas. In general, mean PM concentrations varied among areas but without a consistent pattern. The observed changes in both PM fractions were more closely related to local traffic conditions—specifically, the occurrence of traffic jams, intersections of major roads, and the presence of traffic lights—as reflected by the number of peaks (Table S5).

The mean traffic-background values for PM2.5 and PM10 (Table S5, columns 11 and 12, respectively) exhibited substantial variation depending on the measurement day. A comparison between traffic-background data and the average PM concentrations recorded by the five stationary stations of the Warsaw Air Quality Monitoring Network (Fig. S2; Table S3) revealed strong positive linear correlations, with Pearson coefficients ranging from 0.64 to 0.88 for PM2.5 and 0.87 to 0.92 for PM10. This indicates that the overall pollution level in the city significantly influences PM concentrations recorded along roads. Furthermore, the mean PM2.5 and PM10 concentrations recorded along any route were generally higher than the corresponding mean PM concentrations measured at stationary monitoring stations, except at the urban-traffic station located on Niepodległości Street in the city center, where traffic density is particularly high during rush hours. This is expected, as background stations are located farther from main roads, while suburban-traffic stations are only moderately affected by vehicle emissions. In contrast, urban-traffic stations situated near busy roads show much smaller differences compared with in situ measurements (Tables S3, Table S5).

On July 18, a.m., the average traffic background concentrations of PM2.5 and PM10 across all areas were approximately 23.6 and 25.2 μg/m^3^, respectively (Table S5). In comparison, background stations recorded PM2.5 and PM10 concentrations ranging from 4.7 to 7.6 and 7.7 to 12.1 μg/m^3^, respectively. During the same period, the urban-traffic station recorded PM10 and PM2.5 concentrations of 22.3 and 8.9 μg/m^3^, respectively (Table S3). These findings suggest that in situ PM measurements along heavily trafficked roads represent a combined effect of the citywide PM background level and the additional contribution from road traffic. The citywide background effect is significant because daily fluctuations in PM levels can substantially amplify the concentrations recorded near roads. Consequently, the exposure of pedestrians traveling along busy streets to respirable PM fractions is likely higher than what stationary monitoring stations indicate. This is particularly evident in the consistently higher PM2.5 concentrations measured along roads compared to those recorded by stationary stations. Consequently, pedestrians moving along these roads are more vulnerable to the respirable fraction of PM and the diseases it causes.

The contribution of the coarser fraction (i.e., PM10–PM2.5, to PM10) to total PM₁₀ was found to be much higher in peak conditions than in traffic background situations (Table S6). This finding suggests that the abundance of the coarse fraction may result from specific vehicle movements at intersections, traffic lights, and pedestrian crossings, as well as during traffic congestion. The frequent deceleration and acceleration of vehicles can significantly influence the fuel combustion process within engines. Lower engine speeds during slow-moving traffic and higher speeds during acceleration can lead to more polluted exhaust emissions and increased fuel consumption. These fluctuations can markedly alter the proportion of emitted PM fractions, thereby changing the contribution of coarse and fine particles to total PM. Because the (PM10–PM2.5)/PM10 parameter is independent of total concentration, it reflects only the relationship between the two fractions. Thus, when this parameter correlates with traffic intensity, it indicates that the sources of PM emissions differ between heavy and light traffic conditions. However, nonexhaust emissions arising from brake wear, tire wear, and road surface abrasion should also be considered additional sources of coarse PM. According to Philippe et al. (2021), brake wear contributes approximately 21% of traffic-related PM10 in urban areas and accounts for 16–55% by mass of total non-exhaust PM10 emissions in such environments (Harrison et al. 2021). Typically, PM10 exhibits a unimodal mass size distribution, with a peak between 2 and 6 μm. On average, the PM10 emission factor is estimated at 6.7 mg km⁻^1^ vehicle⁻^1^ (Bessagnet et al. 2022).

Finally, the higher concentrations of PM2.5 measured near roads compared with those recorded at stationary monitoring stations suggest a relatively high health risk for pedestrians moving along roadways—particularly at intersections and in areas with frequent traffic congestion—due to the inhalation of elevated levels of fine PM fractions. Our procedure for determining PM concentrations directly within traffic was designed to evaluate the potential health effects of PM exposure under conditions where large numbers of pedestrians and cyclists are present near roads during rush hours. This approach aligns with the methodology of Cipoli et al. (2022), who monitored PM concentrations in areas with substantial pedestrian and bicycle activity. Their study found that PM originating from vehicle traffic significantly affected even pathways in parks located farther from main roads.

We also considered whether the measuring routine might influence the results. While the monitoring car traveled on the bus line with right-side traffic and at varying speeds due to road conditions (occasionally braking and stopping), its movement was smoother than that on the other, more crowded lines. Higher PM values were recorded before the car was forced to stop due to road conditions (Fig. S5). We also observed some significant PM2.5 peaks during the steady movement of the monitoring car (see Fig. S3). Therefore, we concluded that the car’s speed did not significantly affect the measured PM value.

The intake part of the aerosol monitor, which is located at the passenger window, collects air closer to the right side of the road and pedestrian paths. This helped us monitor the impact of PM emissions on pedestrians. The monitoring vehicle was traveling in the far right lane, with adjacent vehicles approaching only from the left (driver’s side). In this configuration, the impact of air turbulence generated by these vehicles is decreased and has the least impact on PM recording. We also believe that this air collection location is optimal for averaging the readings over short distances.

It should be noted that the PM register in our study represents only measurements obtained from a mobile PM meter. As long as the particle size is within the measurement range (up to PM10), exhaust and non-exhaust emission sources cannot be differentiated. In fact, non-exhaust emissions can significantly contribute to PM recordings, particularly near intersections where brakes are used more frequently.

### Health effects of inhaling PM fractions while walking along roads with high-traffic intensity

The assessment of total PM deposition in the HRT, segregated by particle size, is of fundamental importance for studying deposition patterns across lung regions. The distribution of individual PM fraction concentrations in Areas 1–4 exhibited a bimodal pattern, with the predominant mode (88–92%) occurring between 0.1 and 4.0 μm and a secondary mode (8–12%) occurring between 4 and 10 μm. From the perspective of this study, it is therefore particularly relevant to analyze the deposition parameters of the respirable fractions PM1–PM4 and the coarse fraction PM10. The total deposition of PM1 (Fig. 5) decreased with increasing age among the study groups. The highest proportion of the finest particles—approximately 43%—was deposited in the HRT of children aged 3 years, while lower proportions of about 28% and 33% were observed in adults aged 21 and 25–30 years, respectively. Furthermore, approximately 25% of the finest particles were deposited in the URT during a single inhalation cycle, and up to 15% were deposited in the P region.

A quantitative comparison with previous MPPD studies indicates that the trends observed in the present study are consistent with published data. The markedly higher PM10 deposition mass in the TB region of 3-year-old children compared to adults aligned with previous findings wherein coarse particles were observed to deposit more readily in younger age groups due to smaller airway dimensions and greater particle impaction. Manojkumar et al. (2019) reported that PM10 is primarily deposited in the head (55–95%) and the tracheobronchial (3–44%) regions, whereas PM2.5 and PM1 are deposited most heavily in the head (36–63%) and the pulmonary (28.2–52.7%) regions. Similarly, Cipoli et al. (2023) reported TB deposition fractions of PM10 ranging from 4 to 27%, with children being among the most susceptible groups.

The approximately fivefold higher deposited PM10 mass observed in 3-year-old children (9.24 × 10⁻^5^ µg) compared to that in adults (1.38–1.88 × 10⁻^5^ µg) is within the range expected from age-dependent differences in airway geometry reported by previous MPPD simulations. However, absolute deposited masses can vary between studies due to differences in ambient concentrations and breathing parameters.

The result of DMA provides particularly strong evidence for age-related susceptibility. The maximum DMA value for PM10 in the 3-year-old group (0.781 µg m⁻^2^) was more than 30-fold higher than the values obtained for adults (0.019 µg m⁻^2^). This difference is consistent with the finding of previous MPPD-based studies, which demonstrated that children receive substantially greater particle loading per unit respiratory surface area due to their smaller airway dimensions. Fatima et al. (2022) reported that the highest mass flux parameter value was found for 21-month-old children (4.7 × 10^2^ µg/min/m^2^), followed by that for 3-month-old infants (49.2 µg/min/m^2^), whereas the lowest value was noted for 21-year-old adults (6.8 µg/min/m^2^). This finding indicates that babies and children are more vulnerable to PM2.5 pollution than adults during smog. Overall, the numerical differences observed in the present study are not anomalous; rather, they reinforce previous evidence that coarse particles (PM10) deposit preferentially in the tracheobronchial airways of children, whereas finer particles (PM1 and PM2.5) exhibit relatively greater deposition rates in adults due to larger inhaled air volumes and deeper penetration into the respiratory tract.

The removal of fine particles from the alveolar region is typically limited over the exposure period, lasting from several days to up to a month (Patel et al. 2024). This is due to the mechanisms governing particle clearance in the alveoli. The absorption mechanism facilitates PM removal through lymphatic transport or uptake into the bloodstream, whereas the non-absorption mechanism involves the engulfment of particles by macrophages through phagocytosis. The prolonged residence time of particles in the alveolar region allows for their potential translocation into systemic circulation and subsequent distribution to other organs, including the liver, spleen, kidneys, heart, and even the brain (Manigrasso et al. 2017).

Regarding the PM10 fraction (Fig. 5), total deposition was lower in children (0.89) than in adults (0.96). Additionally, the distribution of this fraction varied across HRT regions compared with the respirable fraction (< 4 μm). As previously established, the predominant proportion of PM10 (approximately 57%) was deposited in the URT, with a significant portion (over 25%) in the TB region and a relatively small fraction (1–7%) in the P region. A comparison of deposition patterns for particles < 10 μm among age groups indicated that, during a single inhalation cycle, the TB region is substantially more exposed to coarse PM fractions in children than in adults. Manojkumar et al. (2019) noted that particles deposited in the TB region are cleared relatively quickly—within a few hours—owing to the efficient function of the mucociliary clearance mechanism. When this clearance mechanism is impaired, ciliary movement becomes restricted, leading to mucus accumulation within the respiratory tract. Such inadequate cleansing facilitates microbial proliferation, resulting in inflammation (Pedersen 1990).

In the HRT of adults aged 21–30 years, the least deposited particles are those within the 0.1–1 μm size range (0.28–0.33), whereas the deposition of particles measuring 1–10 μm ranges from 0.73 to 0.96 (Fig. 5). Adults tend to deposit particles between 0.1 and 10 μm primarily in the URT, while in the pulmonary region, the maximum deposition of respirable particles occurs at approximately 2.5 μm. A notable difference is observed when comparing the deposition distribution in 3-year-old children. In this group, the maximum deposition in the P region was recorded for PM4. As demonstrated by Nachman et al. (2016), exposure to elevated concentrations of PM2.5 significantly affects the incidence of circulatory and respiratory illnesses and may also influence fetal development. This can be attributed to the ability of PM2.5 particles to penetrate the lower respiratory tract, subsequently entering the lungs and circulatory system following phagocytosis by macrophages. Numerous epidemiological studies have demonstrated a strong correlation between PM2.5 exposure and an increased risk of mortality from cardiovascular diseases—particularly ischemic heart disease—as well as a higher incidence of neurological disorders, including Parkinson’s disease (Genc et al. 2012). Studies conducted by Gehring et al. (2010) in Europe also revealed a notable association between PM2.5 exposure and the occurrence of respiratory infections in children. Similarly, Karr et al. (2009) reported a potential link between PM2.5 exposure and the development of bronchiolitis in infants.

In adults, the majority of PM10 is deposited in the URT, with less than 1% deposited in the P region and approximately 4% in the TB region. This finding stands in stark contrast to the observations made in 3-year-old children, in whom as much as 25% of PM10 was deposited in the TB region and 7% in the P region. These results suggest that, in 3-year-old children walking along sidewalks adjacent to streets in Area 1, the deeper regions of the HRT—specifically the TB and P regions—are more susceptible to PM10 deposition. Conversely, in adults, the URT is the primary site of coarse particle deposition. The deposition of the finest particles is governed by Brownian diffusion, which enables their deeper penetration into the airways. In contrast, PM10 is predominantly deposited in the upper region of the HRT through mechanisms involving impaction and gravitational sedimentation (Heyder et al. 1980).

The results revealed significant variations in the deposition behavior of fine particles (PM1) and coarse-transitional particles (PM4) across different age groups and respiratory regions. Unlike PM1, which exhibited a clear shift in total deposition patterns with age, PM4 mirrored the distribution pattern of PM2.5 across the individual HRT regions. This observation suggests that, similar to PM2.5, PM4 is sensitive to age-related differences in airway geometry and respiratory parameters within the considered deposition range. However, distinct age-dependent redistribution was observed for PM1: young adults (21–30 years) exhibited higher mass deposition rates than children, reflecting deeper penetration of ultrafine particles into the lungs under adult breathing conditions. Furthermore, regional PM1 deposition patterns differed significantly among adult subgroups, indicating that minor variations in airway structure and ventilation patterns can substantially impact the behavior of fine particles. Overall, these results suggest that PM1 is more dynamically influenced by physiological factors than PM4, while PM4 exhibits relatively consistent regional deposition behavior with PM2.5 across all age groups.

The findings of this study indicate that older vehicles emit substantial amounts of PM, with a dominant fraction of particles ranging between 2.5 and 10 μm. The coarse PM fraction primarily deposits in the URT, whereas the respirable fraction tends to penetrate deeper into the human respiratory tract. The absorption of PM10 particles by the URT can result in symptoms such as coughing, nasal congestion, and an increased susceptibility to infection (Gładka and Zatoński 2016). One of the significant consequences of exposure to air pollution is the development of allergic rhinitis, an inflammatory condition affecting the nasal and sinus mucosa caused by inhalant allergens. The initiation of inflammatory processes is influenced by the presence of heavy metals, microorganisms, and organic compounds associated with PM (Lee et al. 2015). The prevailing hypothesis suggests that exhaust gas particles interact with airborne allergens to form novel allergenic complexes, which are misidentified by immune system cells, thereby triggering allergic responses (Jang 2013; D’Amato et al. 2005). A clear association has been observed between the increasing prevalence of asthma in children and elevated levels of ozone and particulate matter (McConnell et al. 2003). Although the exact relationship between asthma occurrence and rising PM concentrations remains unclear, Anderson et al. (2013) proposed a potential link between the severity of asthma symptoms and PM10 exposure. Furthermore, studies have shown that asthma is more prevalent among residents of urban areas than among those living in rural settings (D'Amato 2008).

### Possible implications of the implementation of Warsaw CTZ regulations on PM concentration—general remarks

Our studies were conducted before the first stage of Warsaw CTZ zone regulations were implemented on July 1, 2024. Our aim did not include the measurements of the PM following the complete implementation of the CTZ in Warsaw after 2032. We have considered that PM monitoring in the initial years of active CTZ (before 2028) would not provide any additional valuable results.

Traffic in Warsaw—particularly in the city center and along major access roads—is heavily influenced by the large number of intersections. Although bus lanes are intended to improve the efficiency of public transport, slow car movement on adjacent lanes and recurring congestion at intersections persist. When fully implemented, the CTZ system may reduce total PM emissions by limiting the number of cars entering the zone and removing older, high-emission vehicles from circulation. However, even as older cars are phased out and newer vehicles emit less PM, the overall number of vehicles in Warsaw may continue to rise. Warsaw’s inhabitants are getting richer and can afford more than one car. They can also live in the suburbs, farther from the city center, and use their cars for travel. With Warsaw's road system, CTZ cannot be the only solution to reduce traffic and problems with intersections and traffic jams in the city. Our results indicate that reducing the overall number of vehicles traveling into the city—particularly toward the city center—could significantly lower PM emissions, especially at intersections and in pedestrian zones adjacent to major roads.

Although the gradual introduction of the CTZ system may be acceptable to drivers, it is unlikely to result in a rapid improvement in air quality. Additional measures should therefore be implemented, including the further development of low-emission public transportation in suburban areas located farther from the city center and the construction of new parking facilities for commuters traveling from outside the city. While Warsaw’s public transportation system is well developed and highly regarded by users, the habit of driving alone to the city center remains widespread. More effective strategies are needed to reduce unnecessary car traffic within the city. One potential approach could involve introducing a congestion charge—similar to London’s—for entering the CTZ or larger areas of Warsaw, with possible discounts for city residents or taxpayers. Implementing such measures would contribute to improving air quality in Warsaw, which is essential for protecting the health and well-being of its inhabitants.

## Conclusion

1. No significant difference was observed in average PM2.5 and PM10 concentrations between the planned CTZ and its access roads. Elevated PM concentrations were primarily associated with disturbances in traffic movement, such as major intersections, traffic lights, pedestrian crossings, and congestion, highlighting the importance of maintaining smooth vehicle flow to reduce PM emissions. The PM10–PM2.5 fraction contributed substantially more to PM10 concentration during peak concentration events than during traffic background conditions, indicating that traffic disruptions are the major source of coarse particle emissions.
2. Mobile measurements revealed that traffic-related PM consists of a relatively stable background level of traffic emissions and short-term concentration peaks generated by slow-moving and congested traffic. PM2.5 concentrations were higher in traffic background than in citywide background measured at stationary monitoring stations, demonstrating the added value of mobile monitoring for assessing near-road exposure.
3. Respiratory exposure showed a strong age-dependent trend. Although inhaled doses of PM1 decreased with age, the deposition of PM10 fraction in the tracheobronchial and pulmonary regions was significantly higher in young children. The highest PM10 deposition mass density area was observed in 3-year-old children, indicating a markedly greater respiratory surface burden and increased susceptibility to lower respiratory tract diseases.
4. The planned CTZ in Warsaw could improve air quality only if it reduces traffic congestion. Restricting the movement of the oldest and most polluting vehicles alone may be insufficient without additional measures to ensure smoother traffic flow.

## Supplementary Information

Below is the link to the electronic supplementary material.

ESM 1 (DOCX 1.06 MB)

## Data Availability

The data underlying this article will be shared on reasonable request to the corresponding author.
