# Supplementary material for: Real-time record of particulate matter along traffic roads and health risk assessment
Source: Environ Sci Pollut Res Int. 2026 Jul 8;33(22):11323–40. doi: 10.1007/s11356-026-38035-w (PMC13415474; doi:10.1007/s11356-026-38035-w)
Supplement: Supplementary file 1 — (DOCX 1.06 MB) [file 11356_2026_38035_MOESM1_ESM.docx]

**Supplementary Material**

Real-time record of PM along the traffic roads and the health risk assessment

Beata Górka-Kostrubiec*, Tomasz Werner

Department of Magnetism, Institute of Geophysics, Polish Academy of Sciences, Warsaw, Poland

***** Correspondence: kostrub@igf.edu.pl (B.G.K.)

**Table S1.** Mean vehicle traffic intensity measured during morning and afternoon rush hours at automatic monitoring points from May to October 2022. Data sourced from: <https://zdm-warszawa.maps.arcgis.com/apps/dashboards/fb6da3d215334a489709f7dc02726311>

| **Area/Street** | | **Average vehicles/hour** | | | |
| --- | --- | --- | --- | --- | --- |
|  |  | **7-8 a.m.** | **4-6 p.m.** | **Average** |  |
| **Area 1** | al. Jerozolimskie | 1302 | 1848.5 | 1575.25 |  |
|  | Prosta-Żelazna |  | 1021 | 1021 |  |
|  | Żelazna-Chmielna | 495 | 475 | 485 |  |
|  | Towarowa | 996 | 623 | 809.5 |  |
| **Area 2** | Radzymińska | 512.5 |  | 512.5 |  |
|  | Jagiellońska |  | 2009.5 | 2009.5 |  |
|  | Wyb. Szczecińskie |  | 1791 | 1791 |  |
| **Area 3** | Górczewska | 773 | 709 | 741 |  |
| **Area 4** | Ostródzka-Głębocka | 1559 | 1447 | 1503 |  |

**Table S2.** Meteorological data for all session days.

| **Data** | **Temperature (°C)** | | | **Precipitation (%)** | | | **Pressure (hPa)** | | **Rainfall** |
| --- | --- | --- | --- | --- | --- | --- | --- | --- | --- |
|  | **7:00-8:00** | **16:00-18:00** | **7:00-8:00** | | **16:00-18:00** | **7:00-8:00** | | **16:00-18:00** |  |
| 23.05.2023 | 15-18 | 24-25 | 80-60 | | 40 | 1002 | | 999 |  |
| 03.07.2023 | 18-20 | 25-27 | 60-50 | | 40-50 | 997 | | 997 |  |
| 04.07.2023 | 20-23 | 33-32 | 60-40 | | 30 | 1000 | | 999 |  |
| 18.07.2023 | 18-20 | 25-23 | 90-80 | | 40 | 1005 | | 1004 | From 3:00 a.m. to 6:00 a.m. |
| 25.07.2023 | 21-23 | 26-24 | 65-60 | | 40-45 | 988 | | 988 |  |

| Date | Hours | PM10 in µg/m^3^ | | | | | PM2.5 µg/m^3^ | | | | | |
| --- | --- | --- | --- | --- | --- | --- | --- | --- | --- | --- | --- | --- |
|  |  | Urban-traffic station  Av. Niepodległości | Background station  Bajkowa Street | Suburban-traffic station  ul. Chrościckiego | Background station  Tołstoja Street | Background station  Wokalna Street | Urban-traffic station  Av. Niepodległości | Background station  Bajkowa Street | Suburban-traffic station | Background station  Tołstoja Street | | Background station  Wokalna Street |
| 23.05.2023 | 4 p.m.-6 p.m. | 29.03 | 16.83 | 16.37 | 23.77 | 15.10 | 12.17 | 6.17 | 6.33 | | 10.87 | 5.27 |
| 03.07.2023 | 7 a.m. -8 a.m. | 53.85 | 21.85 | 20.65 | 25.15 | 19.30 | 16.25 | 9.05 | 8.65 | | 13.35 | 8.65 |
| 03.07.2023 | 4 p.m.-6 p.m. | 52.03 | 21.13 | 18.27 | 27.87 | 20.63 | 17.00 | 8.10 | 7.47 | | 12.33 | 7.77 |
| 04.07.2023 | 7 a.m. -8 a.m. | 92.55 | 31.85 | 29.15 | 27.10 | 24.55 | 21.40 | 11.50 | 8.75 | | 13.35 | 9.00 |
| 18.07.2023 | 7 a.m. -8 a.m. | 22.30 | 9.25 | 7.70 | 12.10 | 7.40 | 8.85 | 4.95 | 5.85 | | 7.60 | 4.65 |
| 18.07.2023 | 4 p.m.-6 p.m. | 22.93 | 8.50 | 7.77 | 9.27 | 6.57 | 6.17 | 2.10 | 2.20 | | 3.03 | 1.77 |
| 25.07.2023 | 4 p.m.-6 p.m. | 42.20 | 13.92 | 15.70 | 28.03 | 12.33 | 11.13 | 3.67 | 4.80 | | 8.70 | 3.93 |

**TableS3.** PM2.5 and PM10 records from stationary PM monitoring stations in Warsaw. Mean values for the selected time periods were calculated.

**Table S4.** Physiological parameters of specific age groups were used for the simulation of deposition.

| Parameter | | | Option / Values | | | |
| --- | --- | --- | --- | --- | --- | --- |
| Airway | | Species | Child limited 3-years | Child limited 21-years | Human limited |  |
| Morphology | | | | | |  |
|  | Model | | Age-Specific 5-Lobe | Age-Specific 5-Lobe | Yeh-Shum 5-lobe |  |
|  | Functional residual capacity volume (ml) | | 48.2 | 2123 | 3300 |  |
|  | Head volume (ml) | | 9.47 | 42.27 | 50.00 |  |
|  | Breathing route | | Nasal | | |  |
| Breathing parameters | | | | | |  |
|  | Tidal volume (ml) | | 123.3 | 477.2 | 625.0 |  |
|  | Breathing frequency (l/min.) | | 24 | 14 | 12 |  |
|  | Inspiratory fraction | | 0.5 | 0.5 | 0.5 |  |
|  | Aerosol concentration | | PM1, PM2.5, PM4, PM10 | | |  |

**Table S5**. Mean values of PM2.5 and PM10 (with corresponding SD, in μg/m³) for all measurement sessions were calculated for each segment of the traveled route. Q3 quartile values were calculated for PM2.5 and PM10 for each road segment. The number of records with PM2.5 and PM10 exceeding Q3 is also listed. The mean values of PM2.5 and PM10 for the sets of PM below Q3 are also shown and are regarded as the background values. SE values (standard error of the mean) are listed for means of PM2.5 and PM10 calculated for Areas in the same column.

| Date | Area | Street | N | Mean of PM2.5  (total) | SD of PM2.5  (total) | Mean of PM10  (total) | SD of PM10  (total) | Q3  For PM2.5/PM10 | Number of values of PM2.5 and PM10>Q3 | Mean of PM2.5 for data  below Q3(backgroud) | Mean of PM10 for data  below Q3  (backgroud) |
| --- | --- | --- | --- | --- | --- | --- | --- | --- | --- | --- | --- |
|  |  |  |  | µg/m^3^ | µg/m^3^ | µg/m^3^ | µg/m^3^ | µg/m^3^ |  | µg/m^3^ | µg/m^3^ |
| (1) | (2) | (3) | (4) | (5) | (6) | (7) | (8) | (9) | (10) | (11) | (12) |
| 23.05.2023 pm  Tuesday | III | Górczewska | 83 | 35.07 | 8.40 | 40.19 | 12.64 | 38/48 | 20/11 | 31.33 | 36.35 |
|  | I | Av. Jerozolimskie | 199 | 37.99 | 19.07 | 42.42 | 27.58 | 39/44 | 47/49 | 32.04 | 33.95 |
|  | I | Prosta-Zelazna-Chmielna | 92 | 33.87 | 7.39 | 40.19 | 12.15 | 35.5/44 | 23/18 | 30.52 | 35.37 |
|  | I | Towarowa | 48 | 36.94 | 11.42 | 43.23 | 14.25 | 38.5/46.5 | 12/12 | 32.19 | 36.81 |
|  | II | Jagiellońska | 140 | 38.46 | 41.85 | 42.89 | 58.27 | 35.5/39 | 35/31 | 32.01 | 34.42 |
|  | II | Wybrzeże Szczecińskie | 171 | 37.68 | 19.77 | 45.73 | 42.38 | 36/42 | 41/35 | 32.19 | 35.26 |
|  |  | **All route** | **733** | **37.45** | **25.32** | **43.46** | **40.46** |  | **178/156** | **31.81** | **35.00** |
|  |  | **Area I** | **339** | **36.72** | **15.77** | **41.93** | **22.65** |  | **SE(0.85)** | **SE(1.23)** | **SE(0.85)** |
|  |  | **Area II** | **311** | **38.03** | **31.61** | **44.45** | **50.09** |  | **SE(1.79)** | **SE(2.84)** | **SE(1.79)** |
|  |  | **Area III** | 83 | 35.07 | 8.40 | 40.19 | 12.64 |  | **SE(0.92)** | **SE(1.39)** | **SE(0.92)** |
| 03.07.2023 am  Monday | IV | Ostródzka-Głębocka | 162 | 33.12 | 6.25 | 37.93 | 9.79 | 34/40 | 40/36 | 30.69 | 34.65 |
|  | II | Radzymińska-Solidarności | 137 | 32.34 | 7.81 | 35.71 | 9.44 | 34/38 | 26/32 | 29.73 | 32.01 |
|  | I | Av. Jerozolimskie | 75 | 37.79 | 10.34 | 46.11 | 17.38 | 41/52 | 18/18 | 32.93 | 38.26 |
|  | I | Prosta-Zelazna-Chmielna | 70 | 29.77 | 4.07 | 33.59 | 7.29 | 31/37 | 16/16 | 27.83 | 30.11 |
|  | I | Towarowa | 56 | 36.63 | 9.46 | 44.32 | 17.61 | 37/45.5 | 13/1410 | 32.54 | 36.76 |
|  | III | Górczewska | 56 | 36.02 | 6.86 | 41.68 | 9.50 | 37.5/45 | 25/19 | 32.83 | 37.49 |
|  |  | **All route** | **556** | 33.68 | 7.89 | 38.96 | 12.38 |  | **127/129** | **30.77** | **34.30** |
|  |  | **Area I** | **201** | 34.67 | 9.11 | 41.25 | 15.72 |  |  | **SE (0.64)** | **SE(1.11)** |
|  |  | **Area II** | **137** | 32.34 | 7.81 | 35.71 | 9.44 |  |  | **SE (0.67)** | **SE (0.81)** |
|  |  | **Area III** | **56** | 36.01 | 6.85 | 41.68 | 9.50 |  |  | **SE (0.92)** | **SE (1.27)** |
|  |  | **Area IV** | **162** | 33.12 | 6.25 | 37.93 | 9.79 |  |  | **SE (0.49)** | **SE (0.77)** |
| 03.07.2023 pm  Monday | III | Górczewska | 88 | 37.14 | 6.75 | 37.14 | 6.75 | 38.5/45.5 | 22/22 | 34.38 | 38.53 |
|  | I | Av. Jerozolimskie | 128 | 37.21 | 11.37 | 42.98 | 16.13 | 40/42.5 | 30/42 | 31.96 | 34.65 |
|  | I | Prosta-Zelazna-Chmielna | 236 | 31.92 | 5.96 | 34.64 | 6.75 | 33/36 | 16/10 | 30.79 | 33.63 |
|  | I | Towarowa | 19 | 48.63 | 25.45 | 63.36 | 25.45 | 45/60 | 13/13 | 35.5 | 40.68 |
|  | II | Jagiellońska | 57 | 48.96 | 57.42 | 65.77 | 86.44 | 46/57 | 13/14 | 34.68 | 41.19 |
|  | II | Wybrzeże Szczecińskie | 105 | 34.80 | 9.87 | 40.26 | 12.68 | 37/45 | 25/19 | 30.96 | 35.72 |
|  |  | **All route** | **633** | 36.23 | 19.97 | 42.01 | 30.03 |  | **119/120** | **31.89** | **35.50** |
|  |  | **Area I** | **383** | 34.51 | 10.58 | 38.85 | 15.45 |  |  | **SE (0.54)** | **SE (0.79)** |
|  |  | **Area II** | **162** | 39.78 | 35.43 | 49.23 | 53.41 |  |  | **SE (2.78)** | **SE (4.20)** |
|  |  | **Area III** | **88** | 37.14 | 6.75 | 42.44 | 8.99 |  |  | **SE (0.72)** | **SE (0.92)** |
| 04.07.2024 am  Tuesday | IV | Ostródzka-Głębocka | 147 | 45.20 | 8.56 | 53.65 | 15.11 | 48/54 | 34/43 | 41.59 | 46.91 |
|  | II | Radzymińska-Solidarności | 174 | 46.09 | 23.08 | 52.42 | 26.56 | 47/55 | 42/43 | 37.14 | 41.55 |
|  | I | Av. Jerozolimskie | 106 | 49.00 | 23.96 | 60.33 | 28.32 | 50/64 | 23/24 | 41.24 | 49.99 |
|  | I | Prosta-Zelazna-Chmielna | 64 | 37.47 | 9.69 | 44.61 | 13.08 | 40.5/49 | 8/6 | 34.54 | 41.19 |
|  | I | Towarowa | 52 | 39.19 | 12.97 | 47.08 | 16.86 | 40/50 | 11/13 | 34.46 | 40.28 |
|  | III | Górczewska | 58 | 38.86 | 16.46 | 45.22 | 18.39 | 42/49 | 14/14 | 33.73 | 38.6 |
|  |  | **All route** | **601** | 44.17 | 18.35 | 52.13 | 22.33 |  | **132/143** | **38.07** | **43.77** |
|  |  | **Area I** | **222** | 43.38 | 19.17 | 52.69 | 24.29 |  |  | **SE (1.28)** | **SE (1.63)** |
|  |  | **Area II** | **174** | 46.09 | 23.08 | 52.42 | 26.56 |  |  | **SE (1.74)** | **SE (2.01)** |
|  |  | **Area III** | **58** | 38.86 | 16.46 | 45.22 | 18.39 |  |  | **SE (2.16)** | **SE (2.42)** |
|  |  | **Area IV** | **147** | 45.20 | 8.56 | 53.65 | 15.11 |  |  | **SE (0.71)** | **SE (1.25)** |
| 18.07.2023 am  Tuesday | IV | Ostródzka-Głębocka | 153 | 24.87 | 4.72 | 26.45 | 5.70 | 25/27 | 34/37 | 23.09 | 24.07 |
|  | II | Radzymińska-Solidarności | 155 | 46.34 | 54.53 | 49.66 | 57.25 | 39/45 | 37/37 | 26.06 | 27.71 |
|  | I | Av. Jerozolimskie | 95 | 28.28 | 9.68 | 33.12 | 15.61 | 32/37 | 23/22 | 23.85 | 26.48 |
|  | I | Towarowa | 54 | 28.13 | 12.60 | 35.58 | 42.80 | 29/36 | 12/11 | 23.02 | 26.09 |
|  | I | Prosta-Zelazna-Chmielna | 64 | 21.56 | 3.59 | 23.50 | 5.73 | 22/24 | 13/15 | 20.29 | 21.31 |
|  | III | Górczewska | 66 | 29.26 | 18.36 | 30.36 | 18.38 | 27/29 | 14/16 | 22.1 | 22.92 |
|  |  | **All route** | **587** | 31.52 | 30.64 | 34.42 | 32.77 |  | **133/138** | **23.55** | **25.18** |
|  |  | **Area I** | **213** | 26.22 | 9.72 | 30.31 | 38.90 |  |  | **SE (0.67)** | **SE (2.67)** |
|  |  | **Area II** | **155** | 46.34 | 54.53 | 49.66 | 57.25 |  |  | **SE (4.38)** | **SE (4.59)** |
|  |  | **Area III** | **66** | 29.25 | 18.36 | 30.36 | 18.38 |  |  | **SE (2.26)** | **SE (2.26)** |
|  |  | **Area IV** | **153** | 24.87 | 4.72 | 26.45 | 5.70 |  |  | **SE (0.38)** | **SE (0.46)** |
| 18.07.2023 pm  Tuesday | III | Górczewska | 89 | 18.49 | 6.83 | 20.18 | 7.53 | 19/21 | 17/21 | 16.47 | 17.57 |
|  | I | Towarowa | 31 | 18.29 | 3.56 | 21.81 | 6.18 | 19/24 | 7/6 | 16.92 | 19.6 |
|  | I | Prosta-Zelazna-Chmielna | 184 | 15.23 | 2.10 | 17.28 | 3.25 | 16/18 | 27/45 | 14.54 | 15.81 |
|  | I | Av. Jerozolimskie | 127 | 23.19 | 29.16 | 28.35 | 44.20 | 23/24 | 30/31 | 15.45 | 16.72 |
|  | II | Wybrzeże Szczecińskie | 103 | 16.21 | 7.21 | 16.97 | 7.40 | 16/17 | 25/25 | 13.35 | 13.97 |
|  | II | Jagiellońska | 59 | 17.61 | 6.21 | 19.15 | 8.05 | 21/23 | 12/12 | 15.11 | 16.06 |
|  |  | **All route** | **593** | 17.99 | 14.54 | 20.46 | 21.54 |  | **118/140** | **15.00** | **16.19** |
|  |  | **Area I** | **342** | 18.46 | 18.21 | 21.80 | 27.53 |  |  | **SE (0.98)** | **SE (1.49)** |
|  |  | **Area II** | **162** | 16.72 | 6.88 | 17.77 | 7.69 |  |  | **SE (0.54)** | **SE (0.60)** |
|  |  | **Area III** | **89** | 18.49 | 6.83 | 20.18 | 7.53 |  |  | **SE (0.72)** | **SE (0.80)** |
| 25.07.2023 pm  Tuesday | III | Górczewska | 74 | 22.53 | 8.51 | 26.00 | 16.29 | 23/25 | 16/18 | 19.45 | 20.95 |
|  | I | Towarowa | 24 | 29.67 | 9.36 | 32.63 | 9.97 | 38.5/40 | 6/6 | 25 | 27.79 |
|  | I | Prosta-Zelazna-Chmielna | 85 | 24.91 | 15.74 | 31.19 | 27.57 | 25/30 | 20/18 | 19.06 | 21.45 |
|  | I | Al. Jerozolimskie | 169 | 23.60 | 13.23 | 25.72 | 14.09 | 26/29 | 35/41 | 18.74 | 19.87 |
|  | II | Wybrzeże Szczecińskie | 88 | 18.44 | 3.56 | 19.42 | 3.78 | 20/22 | 21/18 | 15.07 | 16.03 |
|  | II | Jagiellońska | 62 | 30.37 | 25.26 | 35.53 | 28.61 | 30/38 | 14/15 | 21.4 | 23.4 |
|  | IV | Ostródzka-Głębocka | 37 | 17.65 | 3.34 | 18.76 | 3.49 | 20/21 | 7/8 | 16.3 | 17.28 |
|  |  | **All route** | **539** | 23.46 | 14.00 | 26.55 | 18.54 |  | **119/124** | **18.98** | **20.5** |
|  |  | **Area I** | **278** | 24.52 | 13.84 | 27.99 | 19.16 |  |  | **SE (0.83)** | **SE (1.15)** |
|  |  | **Area II** | **150** | 23.37 | 17.41 | 26.08 | 20.17 |  |  | **SE (1.42)** | **SE (1.65)** |
|  |  | **Area III** | **74** | 22.53 | 8.51 | 26.00 | 16.29 |  |  | **SE (0.99)** | **SE (1.89)** |
|  |  | **Area IV** | **37** | 17.65 | 3.34 | 18.76 | 3.49 |  |  | **SE (0.55)** | **SE (0.57)** |

**Table S6.** Average percentage contribution of fraction PM10–PM2.5 to PM1 and PM2.5 to PM10 for the individual streets in the study areas.

| Contribution of PM fractions for peaks and traffic-background | | |
| --- | --- | --- |
|  | Fraction (PM10-PM2.5) to PM10 | Fraction PM2.5 to PM10 |
| Peaks | 24% | 76% |
| Background | 8% | 92% |
| Contribution of PM fractions for peaks in Area 1 | | |
|  | Fraction (PM10-PM2.5) to PM10 | Fraction PM2.5 to PM10 |
| Av. Jerozolimskie | 25% | 75% |
| Prosta-Żelazna Street | 29% | 71% |
| Żelazna-Chmielna Street | 22% | 78% |
| Towarowa Street | 24% | 76% |
| Contribution of PM fractions for peaks in Area 2 | | |
|  | Fraction (PM10-PM2.5) to PM10 | Fraction PM2.5 to PM10 |
| Radzymińska Street | 19% | 81% |
| Jagiellońska Street | 29% | 71% |
| Wyb. Szczecińskie Street | 28% | 72% |
| Contribution of PM fractions for peaks in Area 3 | | |
|  | Fraction (PM10-PM2.5) to PM10 | Fraction PM2.5 to PM10 |
| Górczewska Street | 21% | 79% |
| Contribution of PM fractions for peaks in Area 4 | | |
|  | Fraction (PM10-PM2.5) to PM10 | Fraction PM2.5 to PM10 |
| Ostródzka-Głębocka Street | 26% | 74% |

**Table S7.** Total and region-specific deposition fractions of PM1, PM2.5, PM4, and PM10 in three different age categories.

| **Regional fraction deposition** | **PM1** | **PM2.5** | **PM4** | **PM10** |
| --- | --- | --- | --- | --- |
| **Average concentrations PM (µg/m^3^)** | **40.45** | **42.14** | **43.83** | **51.04** |
| **Model: Age_Specific-5-Lobe 3 Years** | | | | |
| **URT** | 0.25 | 0.28 | 0.32 | 0.57 |
| **TB** | 0.03 | 0.05 | 0.09 | 0.25 |
| **P** | 0.15 | 0.28 | 0.33 | 0.07 |
| **Total** | 0.43 | 0.62 | 0.73 | 0.89 |
| **Model: Age_Specific-5-Lobe 21 Years** | | | | |
| **URT** | 0.12 | 0.41 | 0.63 | 0.91 |
| **TB** | 0.04 | 0.05 | 0.06 | 0.04 |
| **P** | 0.18 | 0.31 | 0.24 | 0.01 |
| **Total** | 0.33 | 0.77 | 0.93 | 0.96 |
| **Model: Yeh-Shum-5-Lobe 25-30 Years** | | | | |
| **URT** | 0.14 | 0.47 | 0.68 | 0.92 |
| **TB** | 0.04 | 0.06 | 0.07 | 0.03 |
| **P** | 0.10 | 0.20 | 0.16 | 0.01 |
| **Total** | 0.28 | 0.73 | 0.91 | 0.96 |

**Table S8.** Main deposition parameters in the human respiratory tract, including deposition mass, deposition mass per area, and deposition rate of inhaled PM1, PM2.5, PM4, and PM10 fractions in three different age categories.

| **Parameters of fraction deposition** | **PM1** | **PM2.5** | **PM4** | **PM10** |
| --- | --- | --- | --- | --- |
| **Average concentrations PM (µg/m^3^)** | **40.45** | **42.14** | **43.83** | **51.04** |
| **Model: Age_Specific-5-Lobe 3 Years** | | | | |
| **Deposition of mass (µg) x 10^-5^** | 0.62 | 0.88 | 1.27 | 9.24 |
| **Deposition of mass per area (µg/m^2^)** | 0.038 | 0.048 | 0.072 | 0.781 |
| **Mass rate (µg/min) x 10^-4^** | 1.61 | 2.1 | 3.04 | 2.22 |
| **Model: Age_Specific-5-Lobe 21 Years** | | | | |
| **Deposition of mass (µg) x 10^-5^** | 4.08 | 3.8 | 3.33 | 1.88 |
| **Deposition of mass per area (µg/m^2^)** | 0.033 | 0.029 | 0.027 | 0.021 |
| **Mass rate (µg/min) x 10^-4^** | 5.71 | 5.32 | 4.66 | 2.63 |
| **Model: Yeh-Shum-5-Lobe 25-30 Years** | | | | |
| **Deposition of mass (µg) x 10^-5^** | 4.52 | 3.8 | 3.01 | 1.38 |
| **Deposition of mass per area (µg/m^2^)** | 0.058 | 0.049 | 0.039 | 0.019 |
| **Mass rate (µg/min) x 10^-4^** | 5.42 | 4.56 | 3.61 | 1.65 |

**
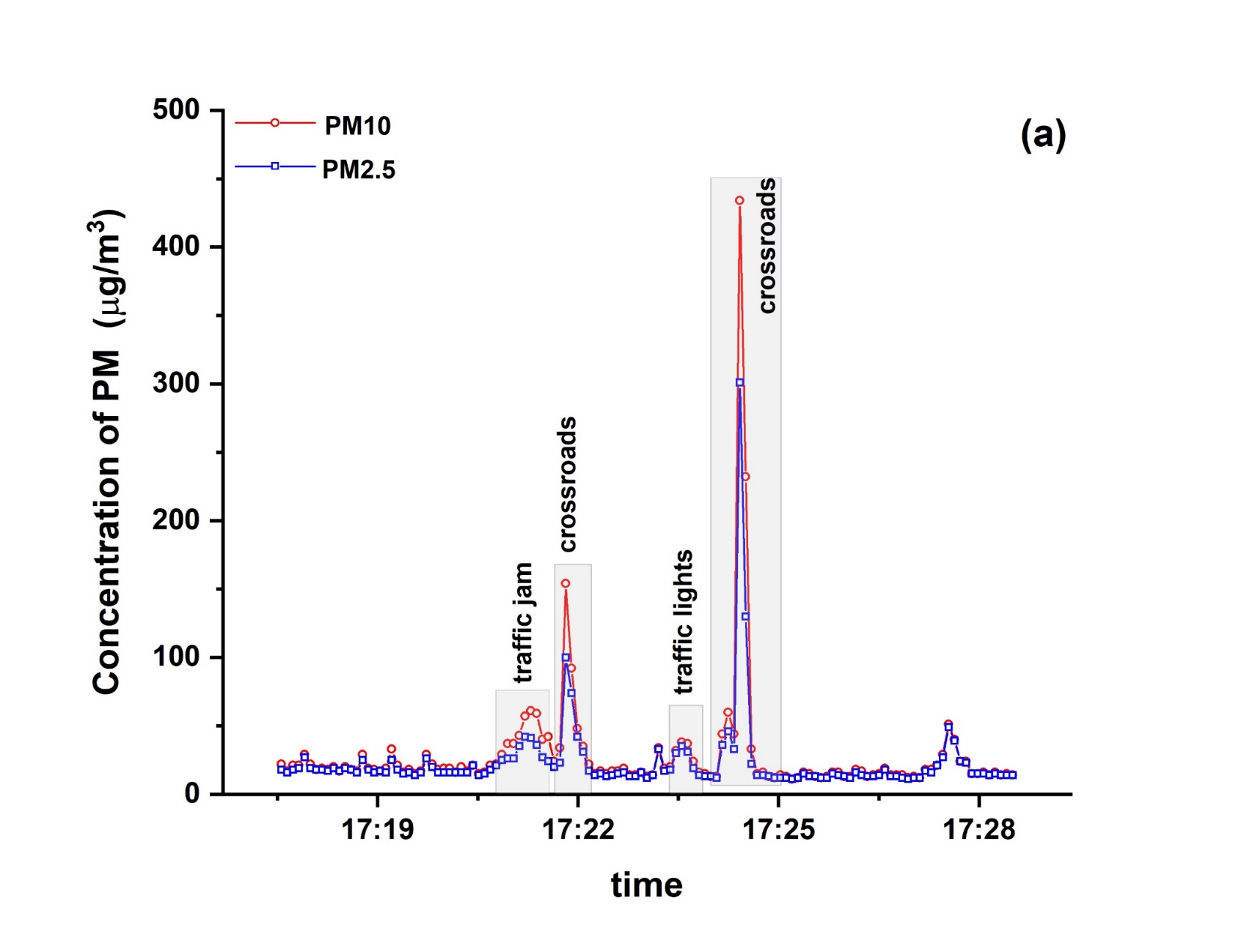
**

**
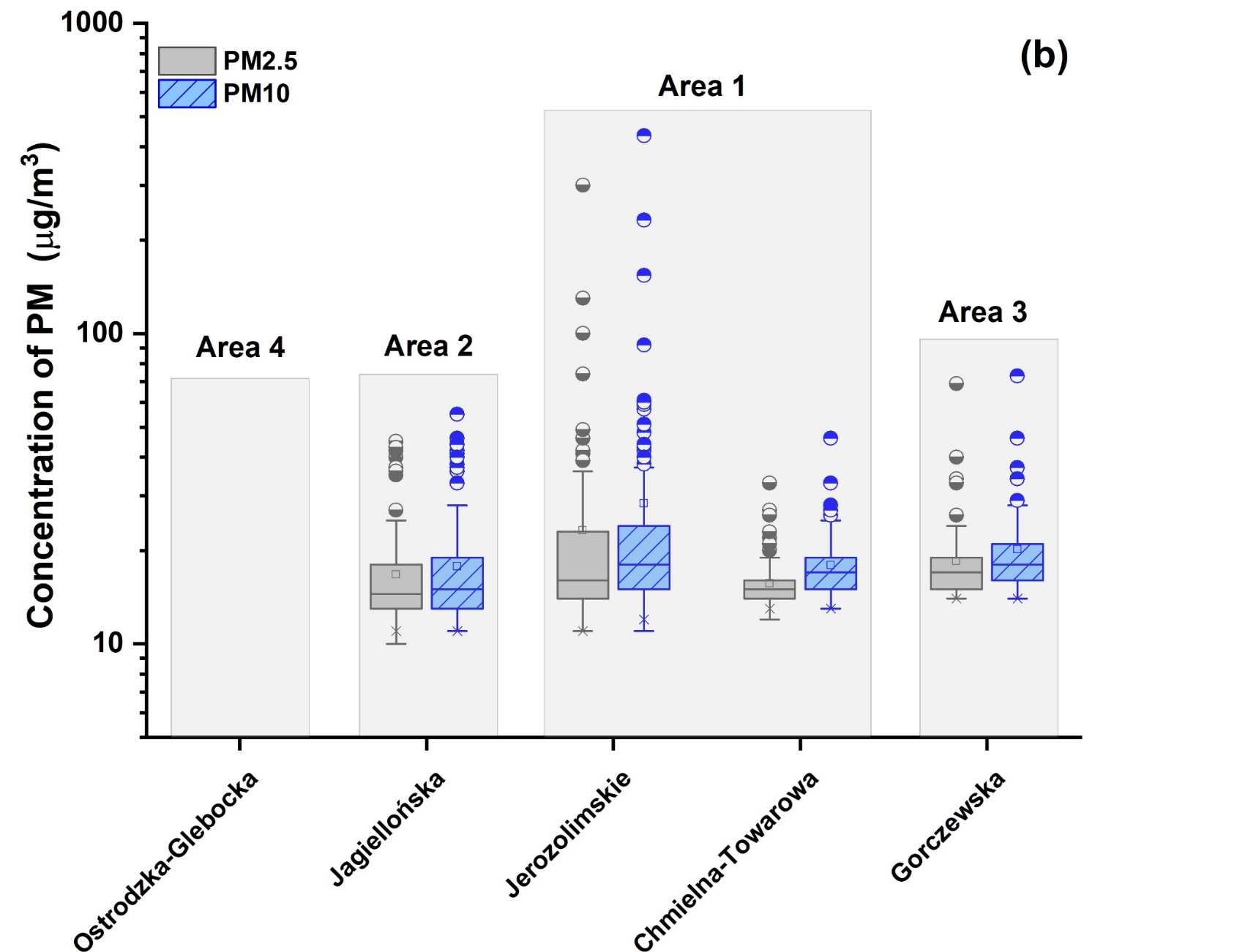
**

**Figure. S1.** ((a) Concentrations of PM10 (red line) and PM2.5 (blue line) along Jerozolimskie Avenue in Area 1. Data were collected on July 18, 2023, during the afternoon rush hour (4:00–6:00 p.m.). The maximum values of PM 10 and PM2.5 were around 450 μg/m³ (more than 30 times above the background level). The electric vehicle traveled 6 km in the bus lane. (b) Box-and-whisker plot showing PM2.5 and PM10 concentrations on roads in all study areas on the afternoon of July 18, 2023. Outliers for each traveled road segment are marked as half-filled circles.


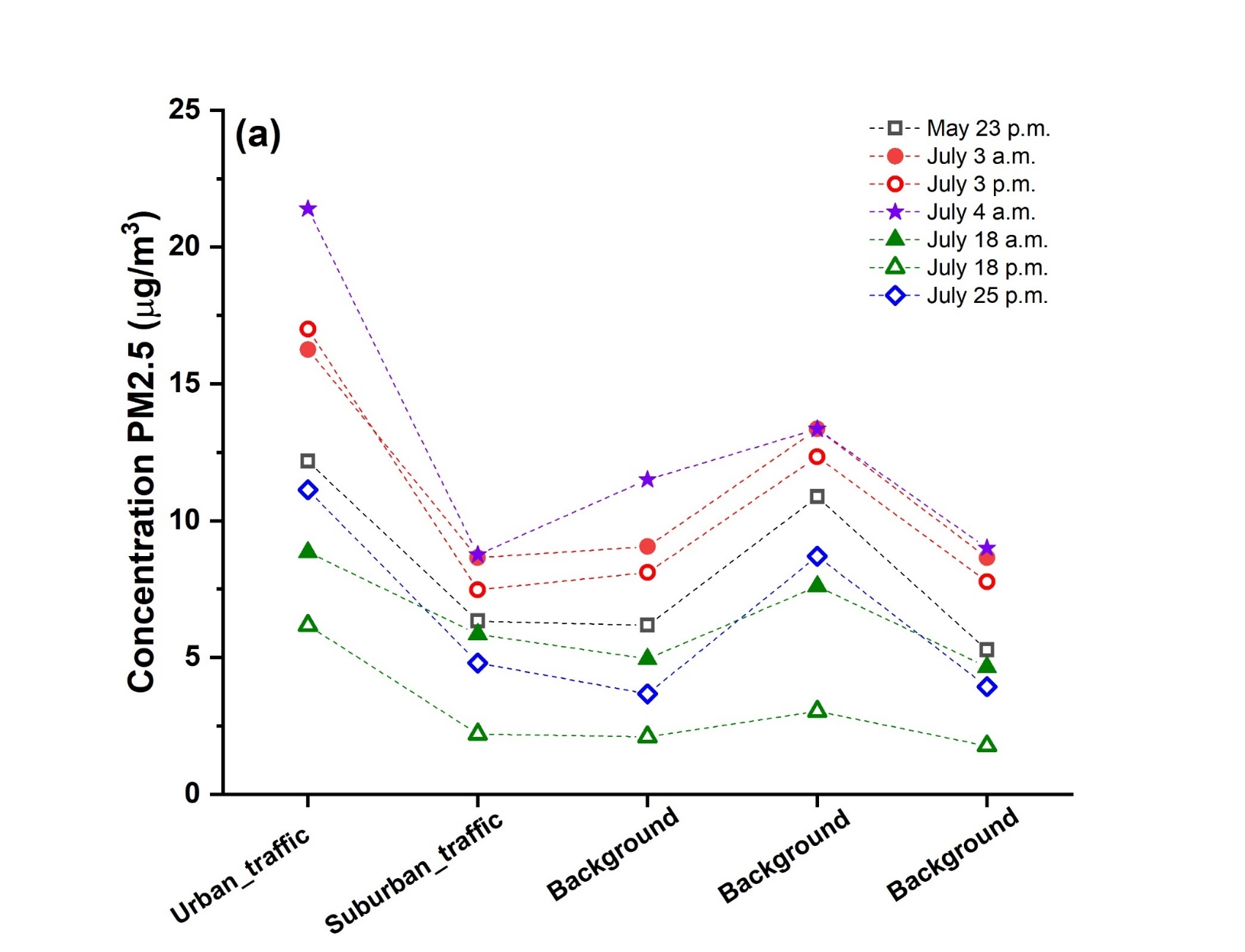

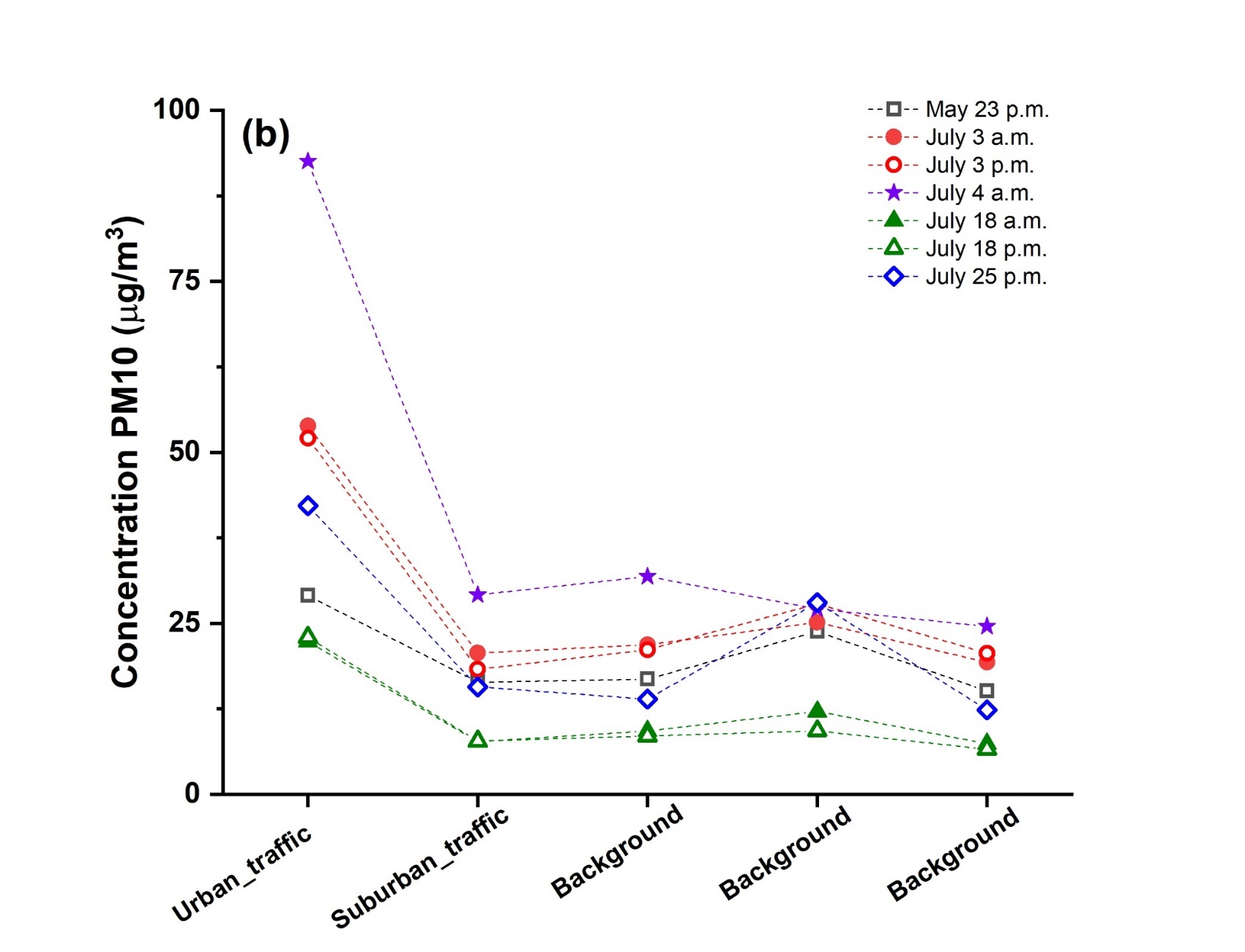


**Figure S2.** Concentration of PM10 and PM2.5 recorded by five stationary monitoring stations; urban-traffic station (Niepodległości Avenue), suburban-traffic station and three background-station. The concentrations of PM were recorded during morning (7-8 a.m.) and afternoon (4-6 p.m.) rush haulers.


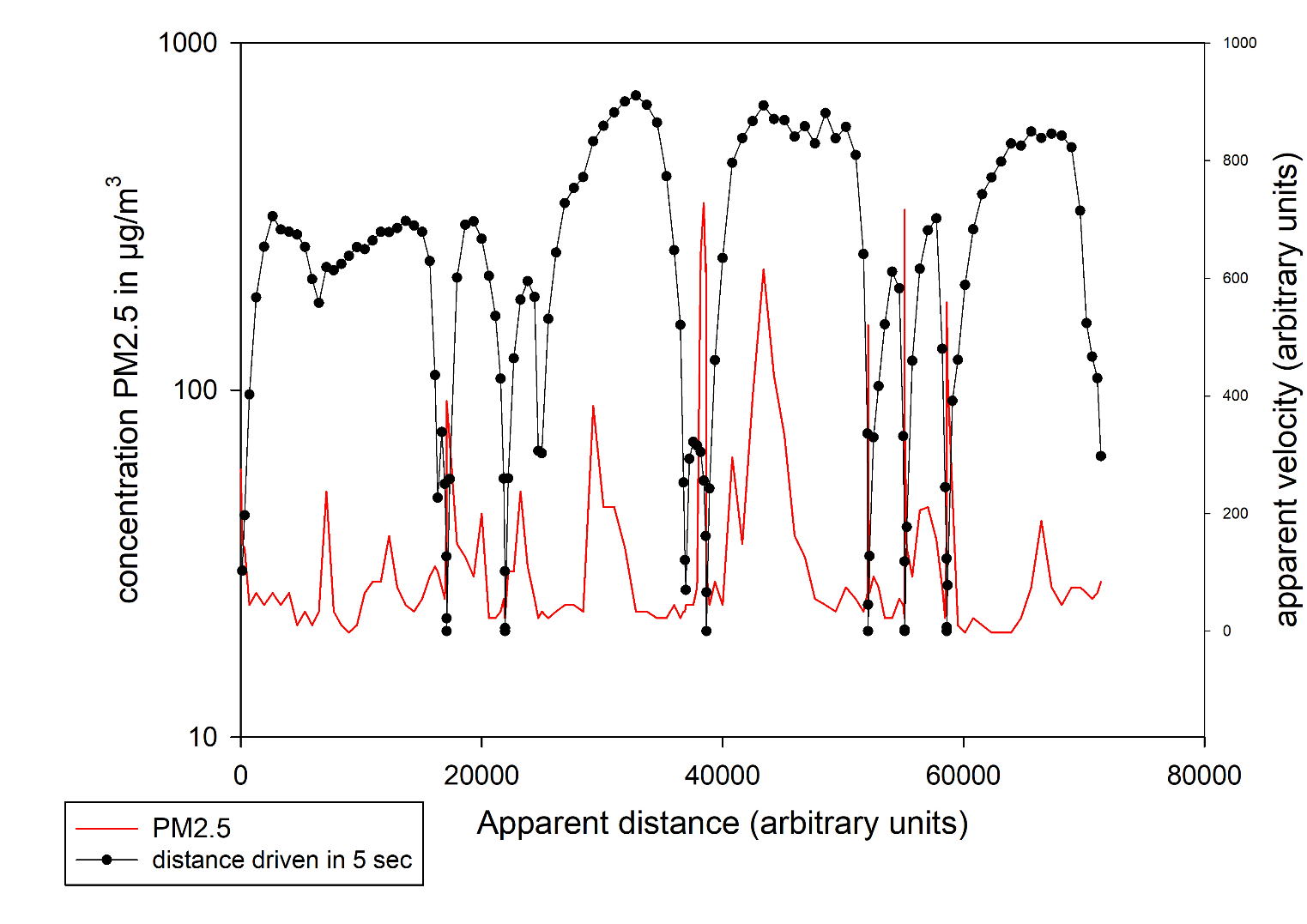
**Figure S3**. The record of PM2.5 concentration along Radzymińska Street in Area 2 (red line). Data were collected on July 18, 2023, during the morning rush hour (7:50–8:02 a.m.) (see also Fig 2a). The record is compared with monitoring device position vs time of measurements that exhibits apparent velocity of the device (black line with dots). Each record was acquired in a 5s long measurement window. For long periods, the speed of the car was kept high and rather steady. In such periods, prominent peaks of PM were observed.
